# Real-time, cross-modal genotype mapping of free-moving *Drosophila* larvae via simultaneous mechano-electrophysiological recording

**DOI:** 10.1126/sciadv.aef7492

**Published:** 2026-09-18

**Authors:** Kairu Dong, Hao Song, Qianhui Zhao, Zhiying Song, Fu Lv, Siouwen Wan, Tianyu Zheng, Yunlong Fan, Wen-Che Liu, Shaomin Zhang, Yongjun Wu, Yuhui Huang, Jizhou Song, Zhefeng Gong, Nenggan Zheng, Kewang Nan

**Affiliations:** ^1^ Qiushi Academy for Advanced Studies, Zhejiang University, Hangzhou 310027, China.; ^2^ State Key Laboratory of Advanced Drug Delivery and Release Systems, School of Pharmacy, Zhejiang University, Hangzhou 310058, China.; ^3^ College of Biomedical Engineering & Instrument Science, Zhejiang University, Hangzhou 310027, China.; ^4^ NHC and CAMS Key Laboratory of Medical Neurobiology, Zhejiang University, Hangzhou 310058, China.; ^5^ Department of Neurology of the Fourth Hospital and School of Brain Science and Brain Medicine, Zhejiang University School of Medicine, Hangzhou 310058, China.; ^6^ Liangzhu Laboratory, MOE Frontier Science Center for Brain Science and Brain-machine Integration, State Key Laboratory of Brain-machine Intelligence, Zhejiang University, Hangzhou 311121, China.; ^7^ State Key Lab of Brain-Machine Intelligence, Zhejiang University, Hangzhou 310058, China.; ^8^ College of Computer Science and Technology, Zhejiang University, Hangzhou 310027, China.; ^9^ School of Materials Science and Engineering, Zhejiang University, Hangzhou 310058, China.; ^10^ Department of Engineering Mechanics, Zhejiang University, Hangzhou 310027, China.

## Abstract

*Drosophila* larvae provide a powerful model for interrogating genes associated with human muscle and neurological disorders; however, existing genotyping and phenotyping approaches remain low-throughput and often rely on destructive, invasive, or toxic procedures. Here, we present a scalable bioelectronic platform that enables real-time, simultaneous mechano-electrophysiological recording from freely moving *Drosophila* larvae in an open three-dimensional (3D) space, allowing high-throughput cross-modal genotype mapping (CMGM). The system integrates conductive and piezoelectric microneedle electrodes into a flexible sensory array that achieves stable, long-term signal acquisition during unrestricted and complex 3D locomotion. By coupling dual-modal signal acquisition with machine-learning-assisted classification, we directly identify muscle defects in unlabeled RNAi-knockdown larvae within 30 minutes, without invasive manipulation or time-consuming sample preparation. Incorporation of both electrophysiological and mechanical waveform features improves overall classification accuracy to 96%, outperforming single-modality approaches. This non-destructive, high-throughput CMGM strategy establishes a generalizable framework for bridging genotype and phenotype in intact, freely behaving organisms, with broad implications for functional genetics and disease modeling.

## INTRODUCTION

As prominent millimeter-scale model organisms for genetic studies (*1*–*3*), *Drosophila* larvae possess conserved genes relevant to human muscle (*4*–*6*) and neurological diseases (*7*, *8*), making them valuable for linking genetic perturbations to sensorimotor phenotypes. For example, the *Gal4-UAS-RNAi* system is widely used for tissue-specific gene knockdown because it avoids the lethality or severe developmental defects often caused by systemic gene disruption, thereby preserving animals for phenotypic analysis (*9*, *10*). When combined with behavioral quantification or GCaMP-based calcium imaging, this strategy has enabled the identification of genetic regulators of motor control, nocifensive behavior, and hyperalgesia (*10*–*14*). However, transgenic RNAi can show variable knockdown efficacy, with many lines failing to achieve robust target suppression (*15*). Accurate genotype identification is therefore essential for connecting genetic perturbations with developmental, physiological, and behavioral phenotypes, ensuring the reproducibility of reverse genetic studies, and supporting genotype discovery and inference in forward genetics (*16*–*25*).

However, existing methods for genotype identification and phenotypic characterization in *Drosophila* larvae remain limited in their ability to provide efficient, real-time functional readouts during free behavior (*21*). Conventional screening strategies often rely on easily identifiable external or developmental features, such as lethality, body size, or color (*26*–*28*), which provide limited insight into dynamic neuromuscular function. For more specific phenotypes, larval studies frequently depend on imaging-based assays that require dissected or chemically labeled samples, followed by image acquisition and manual analysis (*29*, *30*). These workflows are often destructive or toxic, low-throughput, labor-intensive, and technically demanding, thereby limiting their utility for efficiently establishing genotype-phenotype relationships. These limitations highlight the need for non-destructive, high-throughput approaches capable of capturing functional phenotypes from freely moving larvae in real time.

Cross-modal genotype mapping (CMGM) presents a promising opportunity to integrate genomics methods with multimodal sensing technologies (*31*–*33*), which can bridge functional and molecular analyses of genotypes. Data encompassing both cellular and organism levels can be utilized to elucidate the complex functions and dynamic changes of the genome across diverse organism types (*34*–*36*). Previous reports have successfully demonstrated the CMGM approach on mammalian cells via integration of single-cell transcriptomics and bio-electronics (*31*). The existing paired identification of molecular and functional characteristics, however, is primarily applicable at the cellular level. *Drosophila* larvae, on the other hand, exhibit significantly more complex three-dimensional (3D) motion patterns, including head swing, head up, tail swing, and rolling, due to patterned muscle contractions that move stereotypically along the body segments (*37*, *38*). Consequently, analyses at the organism level, encompassing diverse motion patterns, necessitate conditions that permit spontaneous locomotion within an open 3D space. Such requirements are presently incompatible with traditional electrophysiological recording electronics for millimeter-scale organisms, which primarily rely on microelectrode arrays or patch clamping (*5*, *39*–*41*).

Moreover, evaluation of real-time mechanical signals can provide further information on the contact state at the larva-electronics interface and larval movements. Nevertheless, simultaneous mechano-electrophysiological recording is most applicable for organoids, which exhibit little to zero movements (*42*). For other multimodal recording methods of millimeter-scale animals, the animal is either trapped in a narrow micro-channel or limited to a certain scope of in-plane activities (*2*, *43*–*45*).

**Fig. 1.**
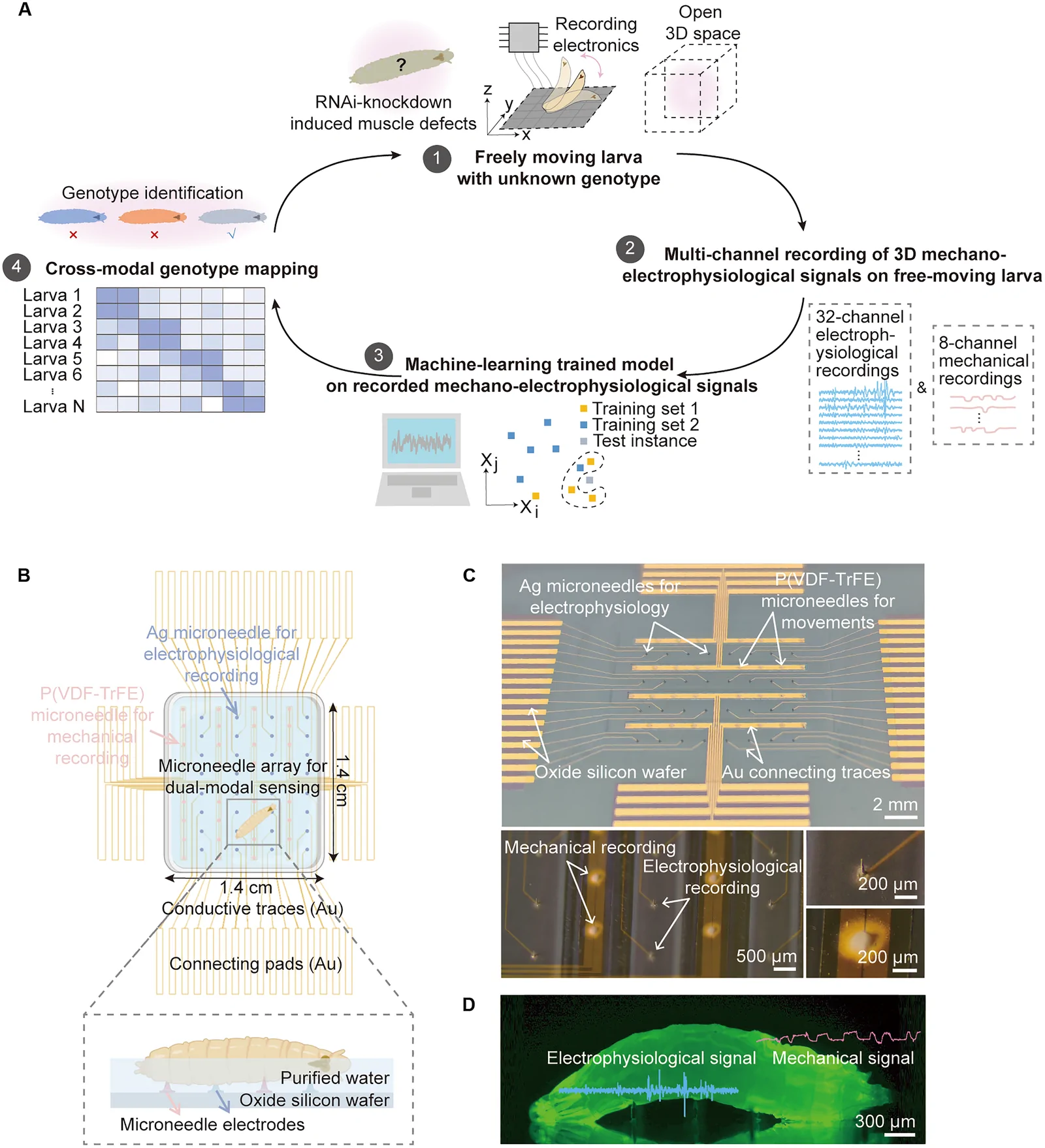
Mechano-electrophysiological recording based cross-modal genotype mapping platform for freely moving *Drosophila* larvae. (**A**) Schematic of four steps in the non-destructive genotype mapping process for *Drosophila* larvae with RNAi-knockdown induced muscle defects, holding promise in related disease diagnosis and treatment. Created in BioRender. Dong, K. (2026) https://BioRender.com/fnnb2wx. (**B**) Plan and section structural diagram of the device used for mechano-electrophysiological recording of free-moving *Drosophila* larvae in an open 3D space. Created in BioRender. Dong, K. (2026) https://BioRender.com/xy5fd7p. (**C**) Optical image of the recording device, with enlarged views of the microneedles for electrophysiology and movement, respectively. (**D**) Illustration of the synchronous use of the mechano-electrophysiological recording and calcium imaging for a *Drosophila* larva.

Here, we report a non-destructive and high-throughput strategy for the CMGM of *Drosophila* larvae (Fig. 1A), enabled by a scalable, real-time mechano-electrophysiological recording platform that permits unrestricted larval locomotion within an open three-dimensional (3D) space. The platform supports a recording area of up to 1.4 × 1.4 cm^2^, accommodating complex and spontaneous larval behaviors without physical confinement (Fig. 1B). To simultaneously interrogate neuromuscular electrical activity and mechanical motion, we integrate two complementary types of microneedle electrodes that interface directly with the larva-electrode contact region (Fig. 1C). Through optimized front-end circuit design and electromagnetic shielding strategies (*46*–*49*), the system achieves high-signal-to-noise mechano-electrophysiological recordings, as validated during benchmark calcium imaging experiments (Fig. 1D). Quantitative features extracted from the dual-modal signals were subsequently used to train a K-nearest neighbors (KNN) classifier, with model performance evaluated by fivefold cross-validation. Incorporation of both electrophysiological and mechanical waveform features increased the classification accuracy for identifying unlabeled RNAi-knockdown larvae with muscle defects to 96%, outperforming classifiers based on either signal modality alone.

## RESULTS

**Fig. 2.**
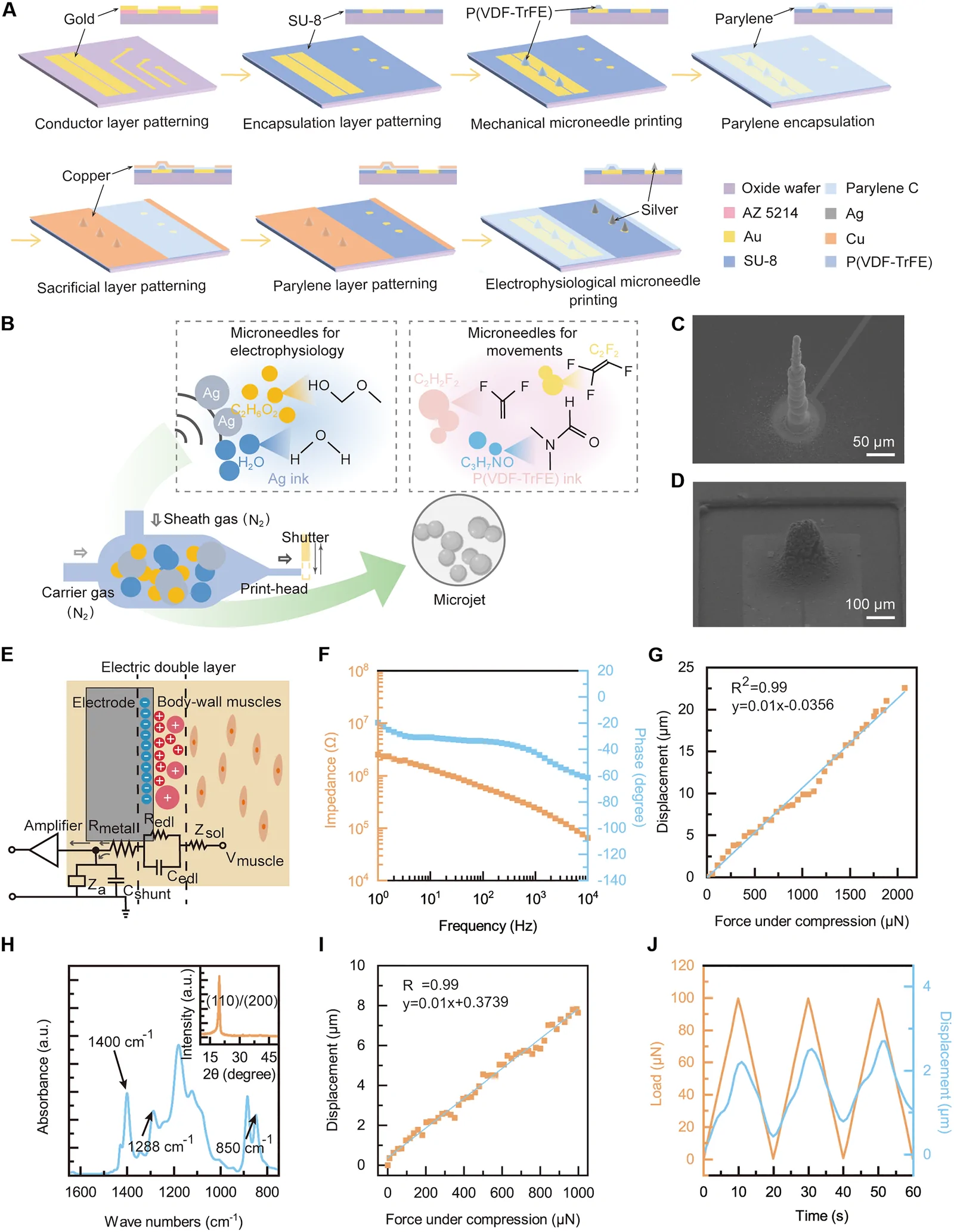
Mechano-electrophysiological recording device for freely moving *Drosophila* larvae. (**A**) Schematic illustration of the process flow for fabricating Poly(vinylidene fluoride-trifluoroethylene) [P(VDF-TrFE)] and silver microneedle array on an oxide silicon wafer substrate. (**B**) Schematic of the formation of the silver and the P(VDF-TrFE) microjet droplets used for printing the microneedle array. (**C**) Scanning electron micrographs (SEM) showing the morphology of a silver microneedle for electrophysiological recording. (**D**) SEM showing the morphology of a P(VDF-TrFE) microneedle for mechanical recording. (**E**) Illustration of the equivalent circuit for electrophysiological signal recording using microelectrodes. (**F**) Electrochemical impedance spectroscopy (EIS) plots for a representative silver microneedle electrode. (**G**) Representative load-displacement plots for the silver microneedle. (**H**) Fourier Transform Infrared (FTIR) spectra and x-ray diffraction (XRD) measurements of the P(VDF-TrFE) composite film (5 wt %). (**I**) Representative load-displacement plots for the P(VDF-TrFE) microneedle. (**J**) Results of the cyclic stress test for the P(VDF-TrFE) microneedle.

As shown in Fig. 2A, we developed a fabrication process for a device enabling simultaneous mechano-electrophysiological recording. The device integrated silver microneedles and Poly(vinylidene fluoride-trifluoroethylene) [P(VDF-TrFE)] microneedles in a staggered distribution on an oxide silicon wafer. Fabrication began with patterning bottom conductive interconnects by thermally evaporating gold (80 nm) using a lift-off process, allowing precise and customizable electrode layouts. A 40-μm-thick SU-8 insulating layer was subsequently patterned by photolithography to expose the bottom electrode plates for microneedle integration. P(VDF-TrFE) microneedles for mechanical sensing were then conformally printed onto the exposed Au electrodes using aerosol jet printing (Aerosol Jet 200, Optomec).

The entire device was encapsulated with a ∼2-μm parylene C layer deposited by chemical vapor deposition to ensure waterproofing of the piezoelectric elements. A laser-cut polyimide mask was applied, followed by selective deposition of a sacrificial copper layer via magnetron sputtering to protect the P(VDF-TrFE) microneedles while exposing the electrode sites for electrophysiological recording (fig. S1). The parylene C in the exposed regions was etched using inductively coupled plasma, and the copper sacrificial layer was subsequently removed by wet etching. Finally, silver microneedles for electrophysiology were printed onto the exposed electrode plates using aerosol jet printing. This fabrication process was fully compatible with transparent glass substrates, enabling concurrent use with inverted optical microscopy (fig. S2).

Figure 2B illustrates the aerosol jet printing process used to fabricate microneedles for mechanical and electrophysiological recording. The silver ink consisted of glycerol and 2,2’-Oxybisethanol as matrix materials and Ag nanoparticles (30–40 nm, 50 wt %) as conductive fillers. The P(VDF-TrFE) ink was prepared by dissolving P(VDF-TrFE) copolymer powder in N, N-dimethylformamide (DMF) at a polymer concentration of 5 wt %. To ensure stable aerosolization and high printing resolution, the viscosities of both inks were controlled within the range of 4–10 mPa·s.

The inks were first homogenized and atomized via ultrasonic oscillation. A nitrogen carrier gas then transported the aerosolized droplets into the printhead, where continuous aerosol streams with defined dimensions were generated by programmed control of shutter timing and sheath gas flow. Precise adjustment of these parameters enabled the controlled stacking and sintering of droplets into conical microneedle geometries with tunable base diameters and lengths (Fig. 2, C and D, and see Materials and Methods for details). The 32-channel microneedle array exhibited a high degree of morphological uniformity, with nearly identical geometric dimensions across individual microneedles (fig. S3). Quantitative analysis showed that the variations in microneedle height and tip diameter were both below 5%, confirming the high reproducibility and precision of the printing process.

We measured the electrochemical impedance spectra of four randomly selected microneedles from the array, which showed comparable impedance profiles and further confirmed the printing uniformity and consistent electrode-electrolyte interfacial properties across channels (fig. S4). We further systematically tested a range of printing parameters for both silver and P(VDF-TrFE) microneedles and characterized the corresponding geometric dimensions, electrochemical impedance, and mechanical properties (figs. S5 and S6). The relationship between printing parameters and microneedle dimensions is summarized in table S1.

Owing to their microneedle geometry, the electrodes formed a tightly sealed electrode-tissue interface, minimizing electrophysiological signal loss (*50*–*53*). The microneedle electrode impedance was modeled as the solution resistance in series with the parallel combination of the double-layer capacitance and charge-transfer resistance (Fig. 2E). When the magnitude of the microneedle electrode impedance was substantially lower than the input impedance of the amplifier (*Z*_a_), electrophysiological signals from the muscle can be recorded with near-complete amplitude fidelity. Electrochemical impedance spectroscopy (EIS) measurements in phosphate-buffered saline showed that the impedance magnitude of an Ag microneedle was approximately 1.3 MΩ at 10 Hz (Fig. 2F), which was orders of magnitude lower than the input impedance of the amplifier. Moreover, the printed Ag electrodes exhibited phase angles of approximately −20° at 1 Hz and − 30° at 10 Hz (Fig. 2F), indicating a pronounced resistive component, likely arising from the Faradaic Ag/AgCl charge-transfer pathway and the volumetric capacitance of the porous printed structure. These non-polarizable properties effectively suppressed phase shift and signal differentiation within the 1–10 Hz frequency range, thereby preserving the temporal dynamics and waveform morphology of the low-frequency electrophysiological signals. The bending stiffness of a 20-μm-diameter cylindrical Ag microneedle with a 5:1 aspect ratio and a Young’s modulus of ∼5 GPa was estimated to be ∼6.28 μN·m. Mechanical robustness was evaluated using nanoindentation (Agilent Nano Indenter G200). Under a loading rate of ∼22.2 μN/s up to 2 mN, the microneedle tip displacement reached 22 μm (Fig. 2G and fig. S7). Cyclic loading tests up to 500 μN further demonstrated stable mechanical performance over repeated deformation cycles (fig. S7).

For mechanical recording, unlike conventional planar thin-film piezoelectric sensors (*42*, *54*), the high-aspect-ratio microneedle geometry allowed flexible deformation along multiple directions, which was critical for resolving complex, multidirectional forces generated by freely moving larvae (*55*). The piezoelectric sensing structure employed a 20-μm counter-electrode gap, spanned by a P(VDF-TrFE) microneedle with a base diameter of ∼100 μm. This configuration exposed a large volume of P(VDF-TrFE) to the poling electric field, enhancing polarization efficiency. The microneedles were poled by air corona treatment at 15 kV for 30 min (fig. S8), resulting in enhanced piezoelectric sensitivity (fig. S9). To characterize the molecular structure, a P(VDF-TrFE) composite film (5 wt %) was spin-coated and cured at 100°C. Fourier transform infrared (FTIR) spectra (750–1650 cm^−1^) revealed characteristic absorption peaks at 850, 1288, and 1400 cm^−1^, confirming the presence of the β crystalline phase (Fig. 2H) (*54*, *56*). To further investigate the piezoelectric properties, x-ray diffraction (XRD) measurements at 2θ angles ranging from 10° to 50° were conducted to study the crystallography and molecular phase of the nanofiller within the composite film. X-ray diffraction (XRD) analysis further identified a diffraction peak at ∼19.7°, corresponding to overlapping (110) and (200) reflections of the β phase, consistent with the FTIR results (Fig. 2H) (*57*, *58*).

Mechanical tests validated that the formed P(VDF-TrFE) microneedles can withstand a pressure at least ten times greater than the average crawling force of larva (∼10 μN) (*55*), and the pressure-deformation relationship was approximately linear below 1 mN (R^2^ = 0.99) (Fig. 2I). Cyclic loading results showed that under three consecutive 100 μN pressure loads, the deformation trend and magnitude of the P(VDF-TrFE) microneedles remained relatively stable (Fig. 2J).

Building on the validated fabrication and material properties of the microneedle array, we next evaluated the electrical and mechanical recording performance of the integrated platform under controlled biological conditions. One silver microneedle was designated as the reference electrode to maintain a stable electrical potential relative to the recording electrode when not in contact with the larva, while a standard Ag/AgCl electrode served as the ground to stabilize the signal baseline. To suppress electromagnetic interference, the entire recording circuit was enclosed within a Faraday cage, with the system ground of the in vivo multichannel recording system (Cereplex Direct, Blackrock Microsystems) connected to the cage to establish a common reference potential and shunt external noise (fig. S10).

To validate the circuit configuration and shielding strategy, extracellular electrophysiological signals were first recorded from the body-wall muscle of intact *Drosophila* larvae using a commercial platinum wire electrode (30 μm diameter) as a benchmark (fig. S11). The microneedle electrodes were then implemented under identical conditions. With the unfiltered noise baseline maintained below ±30 μV in the absence of larval contact, immediate voltage spikes were detected upon contact with a live larva. As shown in fig. S12, the recorded waveforms exhibited amplitudes of 110–390 μV with an average inter-event interval of ∼350 ms, whereas no such activity was observed when euthanized larvae were measured. Wavelet transform analyses further revealed that the dominant power of the electrophysiological body-wall muscle (EBWM) activity was concentrated in the 1–5 Hz frequency range. Both the temporal and spectral characteristics were consistent with previously reported extracellular local field potentials in larval muscle (*5*), validating the fidelity of the microneedle-based recording.

**Fig. 3.**
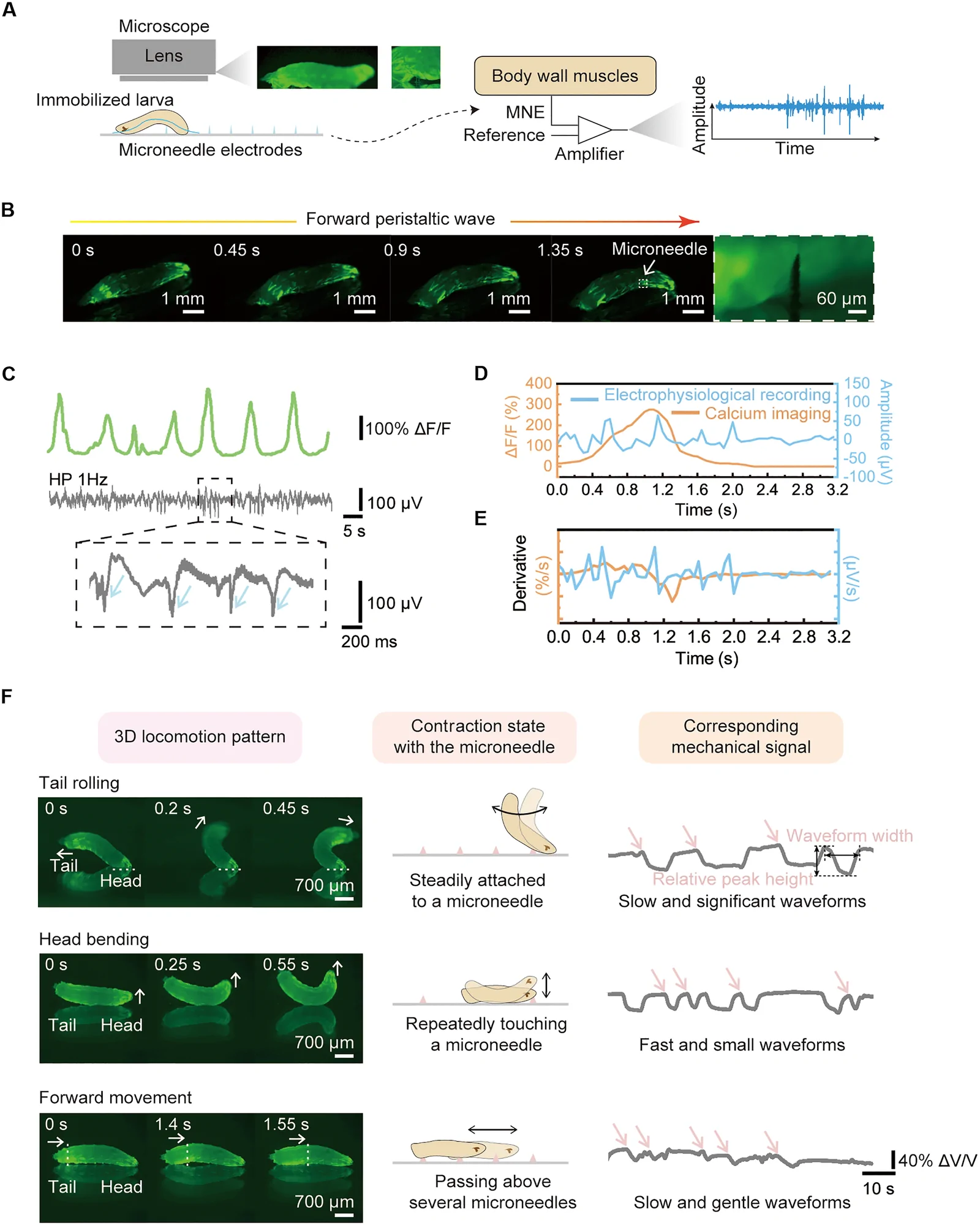
Electrophysiological and mechanical recordings of a *Drosophila* larva combined with calcium imaging. (**A**) Schematic illustration of the electrophysiological recording of an immobilized *Drosophila* larva with the simultaneous calcium imaging measurements. (**B**) Serial images depict changes in calcium ion concentration during the forward peristaltic wave of the larva, which was immobilized against an Ag microneedle electrode. Right: an amplified image shows the larva-microneedle interface. (**C**) Traces, with the same time axis, measured from the larva in (B), show the muscle calcium responses and the synchronous electrophysiological recordings by the Ag microneedle electrode. (**D**) Comparison of amplitude changes of two traces in (C) at the same time scale. (**E**) The derivative of (D) with respect to time. (**F**) Mechanical recordings of three typical movements of a larva by the P(VDF-TrFE) microneedles.

To further correlate the recorded electrical activity with intracellular excitation, we next employed calcium imaging, a widely used method for visualizing calcium transients in muscles and neurons of millimeter-scale organisms (*59*, *60*). *Drosophila* larvae were immobilized against a microneedle electrode fabricated on a transparent quartz substrate (Fig. 3A), enabling simultaneous optical and electrical measurements. Calcium imaging captured propagating peristaltic waves along the body segments, manifested as spatiotemporal fluorescence changes during muscle contraction and relaxation (Fig. 3B). Localized fluorescence modulation was also observed at the microneedle-tissue interface (fig. S13).

To assess whether there was optical interference, electrophysiological noise levels were compared during on and off states of the mercury lamp. The noise baseline was identical before and after turning on the mercury lamp, remaining within ±20 μV in both conditions (fig. S14). Upon lamp activation, the larval muscle contractile activity increased (*61*), calcium concentration changes became more pronounced, and the electrophysiological signal showed more potential fluctuations.

Simultaneous calcium imaging and electrophysiological recordings revealed that individual calcium transients were frequently accompanied by multiple electrical spikes (Fig. 3C). After applying a 1-Hz high-pass Butterworth filter (4th order) to remove low-frequency motion artifacts, the electrophysiological traces exhibited finer temporal structure than the corresponding fluorescence signals. Direct comparison on a shared timescale further highlighted the superior temporal resolution and richer dynamic information captured by the electrical recordings (Fig. 3, D and E).

We further evaluated the mechanical sensing capability of the P(VDF-TrFE) microneedles using *Drosophila* larvae, which exhibit diverse three-dimensional locomotory behaviors (*55*, *62*, *63*), including tail rolling, head bending, and forward crawling owing to their segmented morphology (fig. S15). Using P(VDF-TrFE) microneedles, we successfully characterized the mechanical signatures of these motion types (Fig. 3F). Without calibration against a known force or displacement input, the readout of the mechanical channel can only be interpreted as a relative motion signature. To facilitate relative comparison across different behavioral states, we used the relative peak height (RPH) and waveform width of individual mechanical waveforms as a qualitative feature metric, explicitly noting that these did not correspond to absolute force or displacement values. Tail rolling of the larva produced continuous voltage fluctuations in the microneedle recording; head bending generated rapid, low-RPH oscillations due to repetitive head tapping; forward crawling, on the other hand, induced progressive changes in the waveform width and RPH of signal as peristaltic waves traversed multiple microneedles within a sensing channel (Fig. 3F and fig. S16). These distinct signal features have reflected differences in movement duration and vigor across locomotion modes, demonstrating the potential of our platform to resolve 3D, complex mechanical dynamics in freely behaving larvae.

**Fig. 4.**
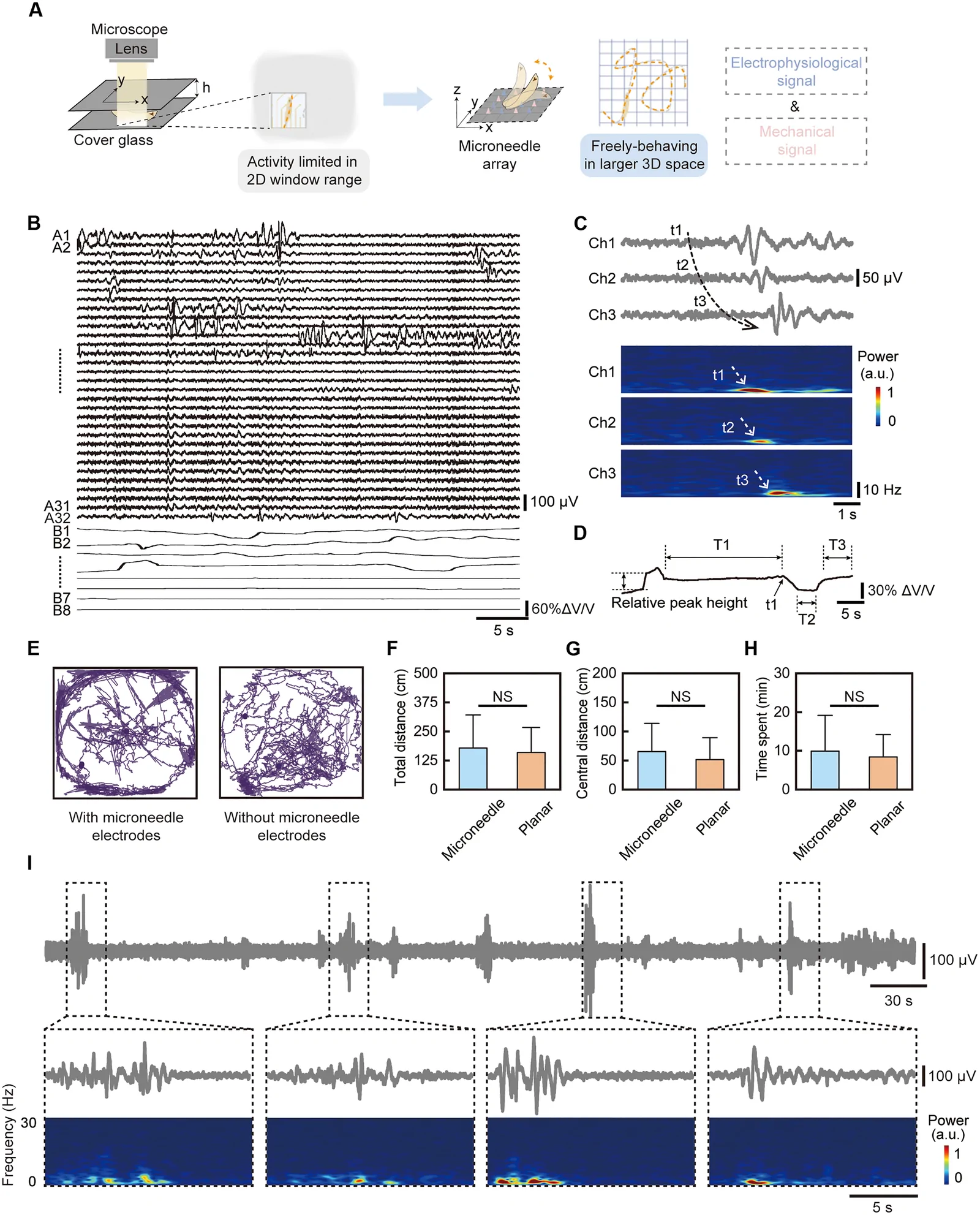
Multi-channel mechano-electrophysiological recordings of free-moving *Drosophila* larvae. (**A**) Illustration of the application of the microneedle array for the mechano-electrophysiological recording of larvae in a free-moving state. (**B**) 32-channel electrophysiological and 8-channel mechanical recordings of freely moving larvae. (**C**) Electrophysiological signals recorded from representative channels with Ag microneedles. (**D**) Mechanical signals recorded from a representative channel with P(VDF-TrFE) microneedles. (**E**) Motion tracking of the freely moving larva in a square open field with and without the microneedle array. Average total distance (**F**), central distance (**G**), and time spent in the central area (**H**) over a 30-min period when freely moving in the 1 cm × 1 cm square field. The values are shown as the mean ± standard deviation (s.d.), *n* = 4. (**I**) Electrophysiological recordings of the free-moving larva during a 450-s period.

Next, we leveraged the on-chip microneedle array to perform recordings from freely moving larvae in an open 3D space (Fig. 4A). In this configuration, the integrated P(VDF-TrFE) microneedles provided a complementary mechanical sensing modality, enriching the functional information accessible during unrestricted behavior. Using this platform, we simultaneously performed 32-channel (A1–A32) electrophysiological and 8-channel (B1–B8) mechanical recording from freely moving larvae (Fig. 4B). Across all channels, the noise baseline remained below ±15 μV, and no detectable electrical cross-talk was observed (Fig. 4B and fig. S17). Time-frequency analyses revealed that electrophysiological signal power remained concentrated in the 1–5 Hz range, consistent with recordings obtained from immobilized larvae (fig. S17).

Notably, compared to manually restrained conditions in Fig. 3, signals from ambulating larvae exhibited reduced random amplitude fluctuations, yielding higher signal fidelity without the need for high-pass filtering. Owing to the mechanical robustness of the microneedles and the minimal force exerted by freely moving larvae, the free-moving recording method effectively reduced the low-frequency baseline noise typically observed under immobilized conditions. Additional high-pass filtering was applied to further ensure the quality of the signals used for analysis. Robust electrophysiological spikes were observed in three consecutive channels with consistent time sequences (Fig. 4C), clearly demonstrating the passage of larva through these microneedle positions.

To determine whether the recorded electrical responses were mechanically driven, we examined experimental paradigms that could selectively suppress endogenous electrical activity while preserving mechanical motion. First, we evaluated whether variations in contact force affected the recorded signals. The loading force was gradually increased from 0 to 1 mN over 90 s, a range that substantially exceeded the normal crawling force of a larva (∼10 μN) (*55*). During this mechanical loading process, the signal recorded by the silver microneedle remained stable, with no detectable potential fluctuation (fig. S18). Second, we used a non-viable larva subjected to externally induced displacement, which allowed endogenous electrical activity to be suppressed while mechanical motion and electrode-tissue contact were maintained (fig. S19). Specifically, the larva was euthanized by immersion in 70°C deionized water for 30 s, placed on the microneedle electrodes, and covered with a coverslip (∼32 mg) to increase the contact area with the recording electrodes. Externally applied displacement through the coverslip generated mechanical motion in the absence of endogenous electrical activity. After 1-Hz high-pass filtering and power-line notch filtering, no detectable potential change was observed (fig. S19).

As a contrasting experiment, we further recorded from live larvae under conditions with minimal mechanical deformation. Signals with waveform characteristics similar to those recorded under freely moving conditions were still detected during pauses in larval contraction (fig. S20). Consistent with recordings obtained with the mercury lamp turned off (fig. S14), fewer electrophysiological events were detected when muscle contraction ceased, which was attributable to the near disappearance of excitatory postsynaptic potentials (EPSPs) in motionless larvae, whereas miniature excitatory postsynaptic potentials (mEPSPs) persisted (*64*). Together, these results indicated that the recorded signals primarily reflected endogenous electrophysiological activity rather than mechanically induced artifacts.

To assess whether our recording method could detect drug-induced neuromuscular responses in larvae, we recorded electrophysiological signals from larvae treated with allyl isothiocyanate (AITC), a potent nociceptive stimulant that elicits vigorous defensive rolling upon topical application. AITC stimulation produced signals with markedly denser spiking activity and substantially larger amplitudes, as captured by the microneedle electrodes (fig. S21). These results demonstrated the ability of our recording platform to detect pharmacologically induced changes in larval neuromuscular activity.

Concurrently, the P(VDF-TrFE) microneedles generated stable and reproducible piezoelectric signals in response to dynamic mechanical stimuli produced during larval movement (Fig. 4, B and D). Following spontaneous charge generation, the RPH of signal decay reflected passive charge leakage through the finite input impedance of the measurement circuit, analogous to a resistance-capacitance (RC) relaxation process rather than active discharge. Accordingly, piezoelectric responses emerged at time points t1, persisted during time intervals T2, and disappeared during T1 and T3 (Fig. 4D).

Owing to the high fabrication yield of 3D printing, the microneedle array exhibited excellent channel-to-channel uniformity in both electrical impedance and piezoelectric sensitivity, ensuring reproducible and consistent mechano-electrophysiological recordings across the centimeter-scale measuring space. For quantitative comparison, signal amplitude was defined as the peak value among all valid waveforms (signal-to-noise ratio > 3:1) recorded within a single channel during a session, and the difference between the peak and minimum amplitudes defined the dynamic range (fig. S22A). Using the minimum amplitude as the reference, 68% of channels met an SNR threshold exceeding 5:1 (fig. S22B).

To assess whether the microneedle array perturbed natural locomotion, we performed an open-field behavioral assay and compared freely moving larvae recorded with the microneedle array to those on planar substrates. Quantitative trajectory analyses (Fig. 4E) revealed no significant differences in total travel distance, center-zone distance, or center-zone dwell time between the two groups (Fig. 4, F to H; *P* > 0.05). No rolling behavior was observed in either the microneedle-present group or the control group. Besides, we quantified the number of backward crawling, left-turning, and right-turning within 10 minutes, indicating minimal behavioral interference (fig. S23). Moreover, the signals associated with drug-induced rolling behavior were clearly distinguishable from those recorded from larvae in a drug-free, freely moving state (fig. S24). This distinction not only indicated that the microneedle electrodes had minimal influence on larval locomotion during signal recording, but also demonstrated the high biocompatibility of the device and recording method, as the electrode interface was not inducing the mechanically evoked rolling escape behavior (*65*). In addition, we used a soft brush to repeatedly bring the larva into proximity with two microneedle electrodes and observed that the larval cuticle indented and rebounded during contact, with no detectable cuticle damage caused by the microneedle electrodes (movie S1). To experimentally validate signal stability, we performed a 450-s electrophysiological recording from a freely moving larva. From the onset of larval contact, the recorded signal, highlighted in the dashed box, exhibited stable and consistent waveforms throughout the experiment (Fig. 4I). Nevertheless, the possibility that larvae do perceive the presence of the microneedles cannot be excluded. Thus, our methodology for mechano-electrophysiological recording of free-moving larvae may only be applicable to conditions in which the larva has intact mechanosensation and neurobehavioral circuits.

**Fig. 5.**
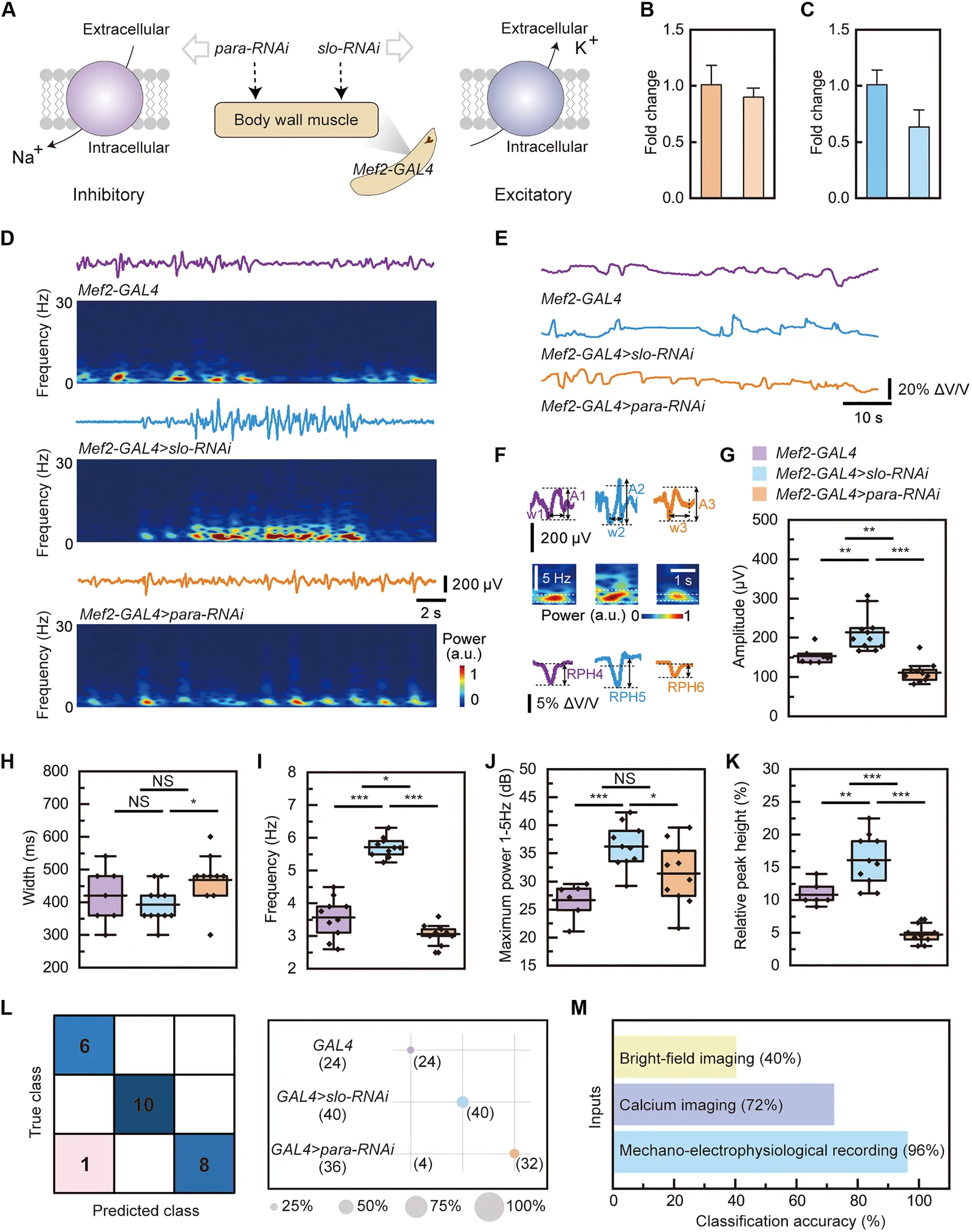
Cross-modal genotype mapping (CMGM) based on the mechano-electrophysiological recordings of free-moving *Drosophila* larvae. (**A**) Illustration of the tissue-specific knockdown of *para* and *slo* in larval musculature and its effects on excitability. Quantification of qRT-PCR results to assess knockdown efficiency of *para* (**B**) and *slo* (**C**) in *Mef2-GAL4 > UAS-RNAi* larvae. Data is presented as the mean ± standard deviation (s.d.), *n* = 3. The recorded electrophysiological signals (**D**) and mechanical signals (**E**) of larvae with three genotypes: the control (*Mef2-GAL4*), *slo* knockdown (*Mef2-GAL4 > UAS-slo-RNAi*), and *para* knockdown (*Mef2-GAL4 > UAS-para-RNAi*). (**F**) Schematic of the characteristics of electrophysiological and mechanical waveforms with annotated duration, amplitude, frequency range, and RPH. The statistical results across three genotypes of amplitude (**G**), width (**H**), frequency (**I**), and normalized magnitude of the wavelet coefficients (**J**) from the electrophysiological waveforms, along with the RPH (**K**) of mechanical waveforms (mean ± s.d.; *P < 0.05; **P < 0.01; ***P < 0.001; NS, not significant; unpaired, two-tailed *t*-tests, *n* = 25). (**L**) CMGM results based on the mechano-electrophysiological recordings of free-moving larvae. (**M**) Classification accuracy of genotype mapping in free-moving larvae based on three independent phenotyping approaches.

Having established stable, high-fidelity mechano-electrophysiological recordings from ambulating larvae, we next evaluated the capability of the platform for cross-modal genotype mapping (CMGM) in a disease-relevant context. Utilizing RNAi technology, which provides a valuable genetic strategy for combining targeted gene regulation with behavioral and physiological phenotyping (*9*, *12*, *13*), we obtained larvae with muscle-specific RNAi-knockdown of ion-channel genes known to modulate membrane excitability. Generally, the phenotypes of RNAi-knockdown lines were relatively subtle compared with those typically observed in mutant lines (*9*). As illustrated in Fig. 5A, knockdown of *para* suppresses sodium influx and reduces cellular excitability, whereas knockdown of *slo* inhibits potassium efflux and increases excitability. The resulting abnormalities in sodium and potassium ion transport are directly manifested in the muscle electrophysiological signals. Using the bipartite *GAL4/UAS* system with the muscle-specific *Mef2-GAL4* driver (*10*, *66*, *67*), we selectively knocked down *para* or *slo* in larval musculature by crossing *Mef2-GAL4* flies with *UAS-para-RNAi* or *UAS-slo-RNAi* lines (fig. S25). Flies carrying only the *Mef2-GAL4* driver served as controls to account for potential effects of *GAL4* expression. Knockdown efficiency was verified by qRT-PCR, which confirmed reduced transcript levels of *para* and *slo* relative to controls (Fig. 5, B to C and fig. S26).

To validate the effectiveness and convenience of our mechano-electrophysiological phenotyping method, we recorded signals from free-moving larvae with muscle-specific RNAi-knockdown. Electrophysiological recordings revealed that knockdown of either gene produced waveforms that were more regular and homogeneous than those of control larvae, indicating increased stability of the excitation-repolarization cycle (Fig. 5D). Consistent with their opposing ionic functions, *slo* knockdown generated sharply peaked waveforms, whereas *para* knockdown resulted in broader, lower-amplitude signals. These differences were further resolved by time-frequency analyses (Fig. 5D). Unlike the relatively uniform electrophysiological waveforms, which were largely decoupled from motion, the mechanical recordings demonstrated high variability associated with distinct locomotor modes. To specifically assess RNAi knockdown-dependent effects on mechanical signals, short-duration and notch-like waveforms with relatively small RPH of similar movements were extracted exclusively (Fig. 5E), thereby controlling for motion-related confounders.

To quantitatively compare genotypes, we extracted key waveform parameters from both modalities, including electrophysiological duration (W1–3), amplitude (A1–3), dominant frequency range (white dotted lines), and relative peak height of mechanical recordings (RPH4–6), as summarized in Fig. 5F. Statistical analyses of these parameters revealed distinct and genotype-dependent mechano-electrophysiological signatures in freely moving larvae (Fig. 5, G to K).

Building on the above identified features, we applied machine learning to evaluate the feasibility of genotype classification based solely on recorded mechano-electrophysiological signals. Waveform characteristics, including duration, amplitude, frequency, wavelet-derived power, and relative peak height, were used as inputs to a conventional K-Nearest Neighbors (KNN) classifier. A dataset comprising 25 independent waveforms from 18 larvae was constructed, including 6 control samples (*Mef2-GAL4*), 10 *slo* knockdown samples (*Mef2-GAL4 > UAS-slo-RNAi*), and 9 *para* knockdown samples (*Mef2-GAL4 > UAS-para-RNAi*). Model performance was evaluated using 5-fold cross-validation. The classifier achieved 84% accuracy using any two electrophysiological features and 88% accuracy when all four electrophysiological features were included (fig. S27). Incorporation of mechanical signal RPH as an additional feature further increased classification accuracy to 96% (fig. S28). The corresponding confusion matrix (Fig. 5L) of the recorded mechano-electrophysiological signatures highlighted the strong discriminability, demonstrating the robustness and necessity of multimodal sensing for accurate genotype mapping.

Finally, we benchmarked the CMGM performance of our platform against established imaging-based behavioral recording approaches, including calcium imaging (fig. S29A and movie S2) and bright-field imaging (fig. S30A and movie S2). For calcium imaging, *UAS-R-GECO1* was co-expressed with the indicated *UAS-RNAi* transgene in larval body-wall muscles under the control of *Mef2-GAL4* to quantify muscle calcium activity after gene knockdown. Consistent with the analysis of mechano-electrophysiological signals, key time-series parameters were extracted from calcium dynamics, including fluorescence intensity, relative fluorescence change, burst duration, and inter-burst interval (fig. S29B).

Under identical imaging settings, the absolute fluorescence intensity of control larvae (*Mef2-GAL4*) was significantly higher than that of the two RNAi-knockdown groups (*Mef2-GAL4 > para-RNAi* and *Mef2-GAL4 > slo-RNAi*), enabling discrimination between control and knockdown larvae. However, calcium imaging failed to distinguish the two RNAi-knockdown strains, despite their opposing muscle electrophysiological abnormalities (fig. S29C). We also performed bright-field crawling-trajectory recordings on 1.5% agar plates and quantified crawling speed as the primary behavioral readout. After 1 min of acclimation before formal recording, bright-field analysis showed substantial inter-individual variability and no statistically significant differences among the three strains (fig. S30B).

We further applied a similar feature-extraction and machine-learning pipeline to the conventional imaging data, using the same KNN model as that used for mechano-electrophysiological recordings (see Materials and Methods for details). The imaging-based dataset included 25 samples from 20 larvae, comprising 10 control samples, 7 *Mef2-GAL4 > UAS-slo-RNAi* samples, and 8 *Mef2-GAL4 > UAS-para-RNAi* samples, constructed from five fluorescence and bright-field imaging features (figs. S29C and S30B). This conventional imaging-based approach achieved a classification accuracy of ∼40% using bright-field imaging data and ∼ 72% using calcium imaging data (fig. S31), markedly lower than the 96% accuracy obtained using our mechano-electrophysiological platform (Fig. 5M).

## DISCUSSION

In this work, we developed a multimodal microneedle electrode platform composed of silver- and P(VDF-TrFE)-based microneedle arrays that enables the simultaneous acquisition of mechanical and electrophysiological signals from freely moving *Drosophila* larvae. By combining this hardware platform with machine-learning-assisted analysis, we established a cross-modal genotype mapping (CMGM) framework that allows muscle defects to be identified in unlabeled larvae in a non-destructive and high-throughput manner. Unlike conventional approaches that rely on immobilization, dissection, or genetically encoded reporters, this strategy directly links emergent biomechanical and bioelectrical phenotypes to underlying genotypes during natural locomotion, thereby preserving physiological relevance while substantially increasing experimental scalability.

Beyond the present demonstration, the CMGM platform offers several opportunities for future development. The microneedle architecture reported in this work is inherently compatible with scalable manufacturing techniques, including photolithography and high-resolution 3D printing, opening a path toward standardized, large-area sensor arrays suitable for population-scale phenotyping. When coupled with state-of-the-art large-scale data acquisition with hundreds or even thousands of channels, such systems could facilitate systematic efforts to bridge genomics and phenomics by leveraging end-to-end deep learning architectures that directly learn discriminative features from raw, multimodal time-series signals, rather than relying on handcrafted descriptors. This data-driven paradigm may enable the discovery of subtle or previously inaccessible genotype-phenotype relationships across diverse genetic perturbations.

Further integration of the mechano-electrophysiological microneedle array with additional modalities, such as calcium imaging, electrical impedance, and biomarker measurements, could extend and enrich the sensing capability across spatial and temporal scales. Such an advanced and sophisticated hybrid sensory platform may provide simultaneous access to biometric information of millimeter-scale animals from subcellular to organism levels, offering a powerful means to interrogate the circuit-level mechanisms that underlie behavior in genetic model organisms. Importantly, because recordings from freely moving larvae do not require immobilization or invasive procedures, the same individual can be monitored longitudinally over extended periods. This feature positions the platform for more demanding applications, including studies of aging, disease progression, or therapeutic interventions that induce gradual and coordinated changes in a range of biometric properties over the lifetime of the animal.

## MATERIALS AND METHODS

### Materials

AZ-5214E photoresist was purchased from Merck. SU-8 2050 photoresist was purchased from MicroChemicals. P(VDF-TrFE) copolymer powder (PVDF/PTrFE = 70/30 mol%, Mw = 450,000 g/mol) was purchased from PiezotechArkema. Silver ink (Metalon, JS-A426) was purchased from NovaCentrix. Parylene C powder was purchased from MaggieNano. All these materials were used directly without any further treatment.

### Fabrication of the SU-8 encapsulated Au conductor

First of all, the oxide silicon substrate was cleaned thoroughly by sonication in acetone, ethanol, and deionized (DI) water. Then, AZ-5214E photoresist was spin-coated over the slides at 500 rpm for 10 s, and 2000 rpm for 40 s successively. After ultraviolet (UV) lithography, the slides were developed to form the microwire layout. Next, thermal vapor deposition was used to deposit a thin bilayer film of Cr (10 nm)/Au (80 nm). Subsequently, the photoresist was lifted off in acetone to obtain the microwire layout. To prepare the encapsulation layer, the SU-8 2050 was then spin-coated over the oxide silicon wafer at 4000 rpm for 40 s, and thermally cured successively at 65°C for 10 min, and 95°C for 30 min. After UV lithography, the oxide silicon wafer after photolithography was transferred onto a hot plate at 65°C for 10 min, and 95°C for 30 min successively. After development for 15 min, the wafer was rinsed with isopropanol and DI water and blown dry. After hardbake at 180°C and natural cooling, the SU-8 encapsulation layer was obtained. The wafer with encapsulated microwire layout was finally cleaned with acetone and sealed in a clean container for later use.

### Fabrication of the microneedle array

We used Ag and P(VDF-TrFE) inks to construct the 3D printed microneedle array. The nanoparticle size in the Ag ink was 30 to 40 nm; the ink viscosity was 4 to 10 cP, and the Ag particle loading in the ink was about 50 ± 5 weight % (wt %). The solvents used for this ink were deionized (DI) water and ethylene glycol. Ethylene glycol acts as a humectant and dispersant to help in the formation of the shank/pillar structures required for this work. The ink used for printing the piezoelectric microneedle was 0.5 g of P(VDF-TrFE) copolymer powder dissolved in 10 mL of N, N-dimethylformamide (DMF) solvent, stirred magnetically for 6 hours at 25°C (*68*, *69*). The solvent was aerosolized during AJ printing via ultrasound energy.

An AJ 3D printer (Aerosol Jet 200, Optomec) was used to print the microneedle array. The AJ system consists of three atomizers (two ultrasonic atomizers and a pneumatic atomizer), a programmable XY motion stage, a light source, and a deposition head. For the construction of the microneedle, we used one ultrasonic atomizer to aerosolize the Ag or P(VDF-TrFE) inks. The aerosolized ink droplets were carried to a printhead by a carrier gas (N_2_). Inside the printhead, the droplets were focused on the nozzle with the help of a sheath gas (also N_2_) to form a microjet. The printing process was carried out by a continuous flow of droplets, which was diverted or resumed by the movement of a shutter. Before printing, the layout of the microneedle array was drawn in AutoCAD using a program in the software AutoLISP (AutoCAD 2015, Autodesk Inc., San Rafael, CA) and converted to a “prg.” file compatible with the printer software. Then, the microneedle was printed by droplet over droplet (*70*, *71*). The diameter of the nozzle used for metals was 100 μm. And the gas flow rate in the AJ machine during printing of Ag microneedle was about 20 sccm (standard cubic centimeters per minute), while that for the sheath gas was about 30 sccm. The diameter of the nozzle used for P(VDF-TrFE) was 200 μm. And the gas flow rate of printing P(VDF-TrFE) microneedle was about 25 sccm, with the sheath gas was about 35 sccm. The platen temperature was kept at 100°C during the printing process.

Printed Ag microneedles on the silicon wafer were sintered at 100°C for 2 hours. The P(VDF-TrFE) microneedles printed on the silicon wafer were sintered at 100°C for 1 hour. The sintering was carried out in a programmable oven with controlled heating rates (Jinghong, DZF-6050).

### Fabrication of the patterned parylene C layer

The deposition of an approximately 2-μm thick parylene C film was carried out in a chemical vapor deposition (CVD) system (MQP-3001 Parylene Coating System). The clean and dry wafer was placed on the sample holder inside the deposition chamber at room temperature. The chamber was initially evacuated to a base pressure below 3 Pa. Parylene C powder was loaded into the evaporation boat, and the evaporator temperature was set at 175°C to sublime the dimer into the vapor phase. The gaseous dimer was then introduced into the pyrolysis zone, where it was cleaved into reactive monomers at a temperature of 680 ± 1°C. Subsequently, the active monomers entered the room-temperature deposition chamber and adsorbed onto the substrate surface, undergoing polymerization and molecular self-assembly to form a uniform polymeric film. Throughout the deposition process, the chamber pressure was maintained at 2–3 Pa for a duration of 4 hours. After the CVD process, a laser-cut patterned polyimide mask (250 μm) was aligned and placed over the parylene C encapsulated wafer. Using the sputter deposition system (DENTON VACUUM DISCOVERY-635), a 150 nm Cu layer was deposited to cover the P(VDF-TrFE) microneedle. Then, the wafer was loaded into an inductively coupled plasma etching (ICP-100A, TAILONG) to etch the parylene C layer that without the protection of the Cu layer. Finally, the sacrificial Cu layer was removed by wet etching.

### Device characterizations

SEM images were obtained using a Zeiss GeminiSEM-560, at an acceleration voltage of 10–15 kV. Electrochemical impedance spectroscopy (EIS) was conducted by an electrochemical workstation (PlamSense EmStat4X) using an Ag/AgCl reference electrode (CHI111) and a 0.3 mm Pt counter electrode. The stress-strain response and fatigue resistance test were measured by the nanoindentation tests (Agilent Nano Indenter G200) using a 1.5 mm by 1.5 mm flat square-shaped indenter probe. The Fourier transform infrared (FTIR) spectra were obtained using Thermo Fisher Scientific Nicolet-iS10. X-Ray Diffraction (XRD) measurements were conducted by Rigaku SmartLab.

### Strains and culturing of *Drosophila* larvae

The Canton-S flies used for mechano-electrophysiological recording (Figs. 3 and 4) were obtained from Bloomington Drosophila Stock Center (BDSC). The RNAi lines (*Mef2-GAL4*, *Mef2-GAL4 > UAS-slo-RNAi*, and *Mef2-GAL4 > UAS-para-RNAi*), used for mechano-electrophysiological recording in Fig. 5, were obtained from the Tsinghua Drosophila Stock Center. Larvae of the genotype *R44H10-lexA; LexAop-GCaMP7s*, used for calcium imaging in Fig. 3, were generated by crossing parental lines obtained from BDSC. Larvae of the genotype *UAS-R-GECO1* were used for calcium imaging of *RNAi* strains were obtained from BDSC. All flies were raised at 25°C on standard medium with a 12-hour light/12-hour dark cycle (*72*). The third-instar larvae were used in all experimental tests. Larvae lacking a locomotor response to brush stimulation were excluded from analysis.

### Electrophysiology measurements of *Drosophila* larvae

The electrophysiological recording was conducted by Blackrock CerePlex Direct voltage amplifier, along with a 32-channel Blackrock μ digital headstage. A flexible anisotropic conductive film (ACF) cable was thermally (150°C, 30 s) bonded to the contact pads of the microneedle array and the other side to the homemade headstage-to-device connector. One of the Ag microneedles in the on-chip array was assigned as the reference electrode, which remained at a fixed potential relative to the recording electrode and was kept out of direct physical contact with the larva, ensuring a stable baseline. A Bayonet Neill-Concelman (BNC) cable was used to connect the low-impedance ground electrode (Ag/AgCl wire, 0.5 mm in diameter) and the headstage-to-device connector, with its outer shield terminated to a Faraday cage for common-mode noise rejection and its center conductor wired to the ground pin of the headstage. The aforementioned on-chip microneedle array, Ag/AgCl ground electrode, and headstage-to-device connector were all placed in the Faraday cage that was connected to the system ground of the Blackrock CerePlex Direct system (fig. S6). Before recording, the microneedle array was immersed in a droplet of deionized (DI) water. This immersion established a stable conductive medium essential for electrophysiological recordings and also provided supplemental isolation against electromagnetic interference. A sampling rate of 2000 samples per second was used. For each larva recording, we picked an individual larva from the standard culture medium and placed it directly on the DI water immersed microneedle array without any cleaning treatment. Recordings were allowed to run for 30 min before being stopped. The primary difficulty in long-duration recordings was larval displacement from the test field. In practice, however, its occurrence was minimal because the surface tension at the droplet edge effectively prevented larvae from leaving the aqueous recording environment.

### Electrophysiological analyses

Matlab codes provided by Blackrock were used to convert raw data files into accessible format. Signal noise was reduced using a combination of a 0.1–1000 Hz band-pass filter and a narrow band-stop filter (49–51 Hz) targeting power-line frequency, both 4th-order Butterworth type. The filtered time-series data were then transferred to Origin for graphing. Spectral analyses (0–30 Hz) were performed using the wavelet transform. The resulting time-frequency data were visualized as heatmaps in MATLAB.

### Piezoelectric recording

Prior to measurements, the P(VDF-TrFE) microneedle array was polarized at room temperature for 30 minutes under a high corona voltage (15 kV) in a corona poling instrument (Balab SPD) to align the electric dipoles in a single direction, optimizing its piezoelectric performance. Subsequently, the electrical signals generated by larval movements were recorded with a programmable electrometer (Keithley 6514), which provided an ultrahigh input resistance of 10 MΩ necessary for detecting weak piezoelectric charges. To effectively suppress power-line interference, the number of power line cycles (NPLC) was set to 1. This setting also provided a suitable measurement rate for capturing the signal waveform.

### Larvae euthanization

A third-instar larva was euthanized with DI water bath at 70°C for 20 min. All other recording conditions were identical to a typical experiment with live animals. The trace in fig. S8 represents the entirety of the data collected.

### Calcium imaging

Calcium imaging of larval body-wall muscles was performed using third-instar larvae expressing the genetically encoded calcium indicator GCaMP7s under the control of the muscle-specific driver *R44H10-LexA*. Fluorescence signals were captured using a stereo microscope with a 2x objective lens (Olympus DFPL) equipped with a filter set (Haokang HZFL-UBG-010-O2) optimized for GCaMP (excitation: 457–492 nm; emission: 505–555 nm). Time-lapse imaging was conducted at a frame rate of 5 Hz to record calcium dynamics.

### Machine learning implementation

Based on the recorded mechano-electrophysiological signals, each sample was represented by five features corresponding to distinct waveform parameters (Fig. 5, G to K) and a categorical label indicating genotype (encoded numerically as 1, 2, or 3). A dataset comprising 25 independent waveform samples across three genotypes derived from 18 larvae was constructed, including 6 control samples (*Mef2-GAL4*), 10 *slo* knockdown samples (*Mef2-GAL4 > UAS-slo-RNAi*), and 9 *para* knockdown samples (*Mef2-GAL4 > UAS-para-RNAi*). Similarly, for the fluorescence and bright-field imaging of freely moving larvae, a dataset of 25 samples from 20 larvae was constructed from five imaging features (figs. S29C and S30B), comprising 10 control samples (*Mef2-GAL4*), 7 *slo* knockdown samples (*Mef2-GAL4 > UAS-slo-RNAi*), and 8 *para* knockdown samples (*Mef2-GAL4 > UAS-para-RNAi*). Then, the dataset was imported into MATLAB, and model training and evaluation were carried out using the built-in Classification Learner App. Considering the limited sample size, the K-nearest neighbors (KNN) model was selected to mitigate the risk of overfitting associated with more complex models. As a "lazy learning" algorithm, KNN does not require the explicit training of intricate parameters and has been shown to exhibit relatively robust performance in small-sample settings (*73*–*75*). Model performance was assessed via 5-fold cross-validation.

### Statistical analysis

The statistics were calculated with Origin (https://www.originlab.com/). For all the experiments, statistical significance between groups was determined using unpaired, two-tailed *t* tests (mean ± s.e.m.; *P < 0.05; **P < 0.01; ***P < 0.001; NS, not significant).
