# Supplementary material for: Real-time, cross-modal genotype mapping of free-moving Drosophila larvae via simultaneous mechano-electrophysiological recording
Source: Sci Adv. 2026 Sep 18;12(38):eaef7492. doi: 10.1126/sciadv.aef7492 (PMC13588200; doi:10.1126/sciadv.aef7492)
Supplement: Supplementary file 1 [file sciadv.aef7492_sm.pdf]

Science Advances
AAAS

Supplementary Materials for

Real-time, cross-modal genotype mapping of free-moving *Drosophila* larvae via simultaneous mechano-electrophysiological recording

Kairu Dong *et al.*

Corresponding author: Nenggan Zheng, zng@cs.zju.edu.cn; Kewang Nan, knan@zju.edu.cn

Sci. Adv. **12**, eaef7492 (2026)
DOI: 10.1126/sciadv.aef7492

The PDF file includes:

Figs. S1 to S31
Table S1
Legends for movies S1 and S2

Other Supplementary Material for this manuscript includes the following:

Movies S1 and S2

Supplementary material

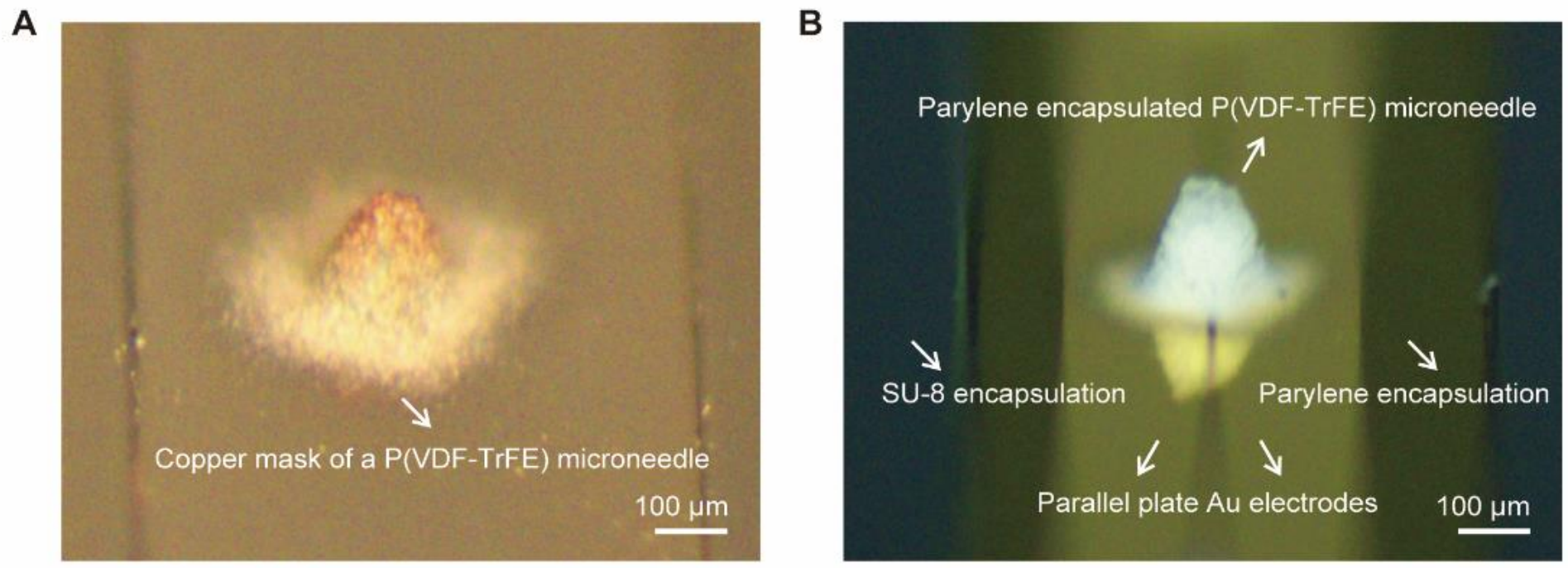

Figure S1. Images of a P(VDF-TrFE) microneedle for mechanical recording. The piezoelectric microneedle with (**A**) and without (**B**) a sacrificial coating layer (Cu, 150 nm).

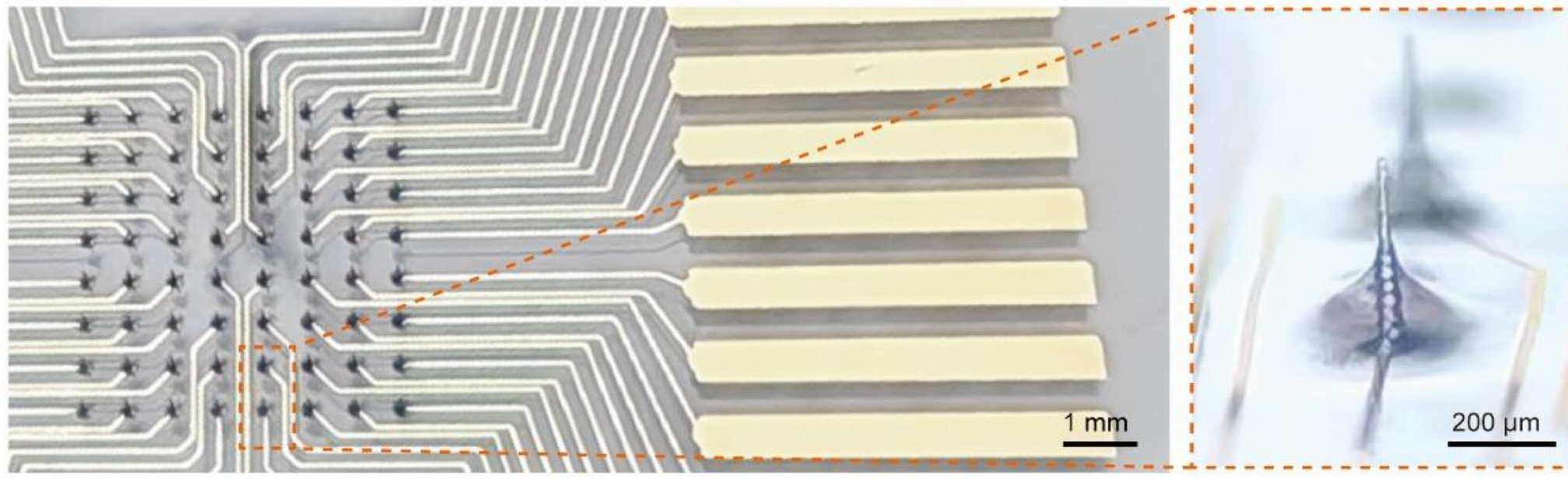

Figure S2. Optical images of a 64-channel microneedle array fabricated on the glass substrate for electrophysiological recording, which is compatible with the simultaneous performance of fluorescence microscopy using an inverted microscope.

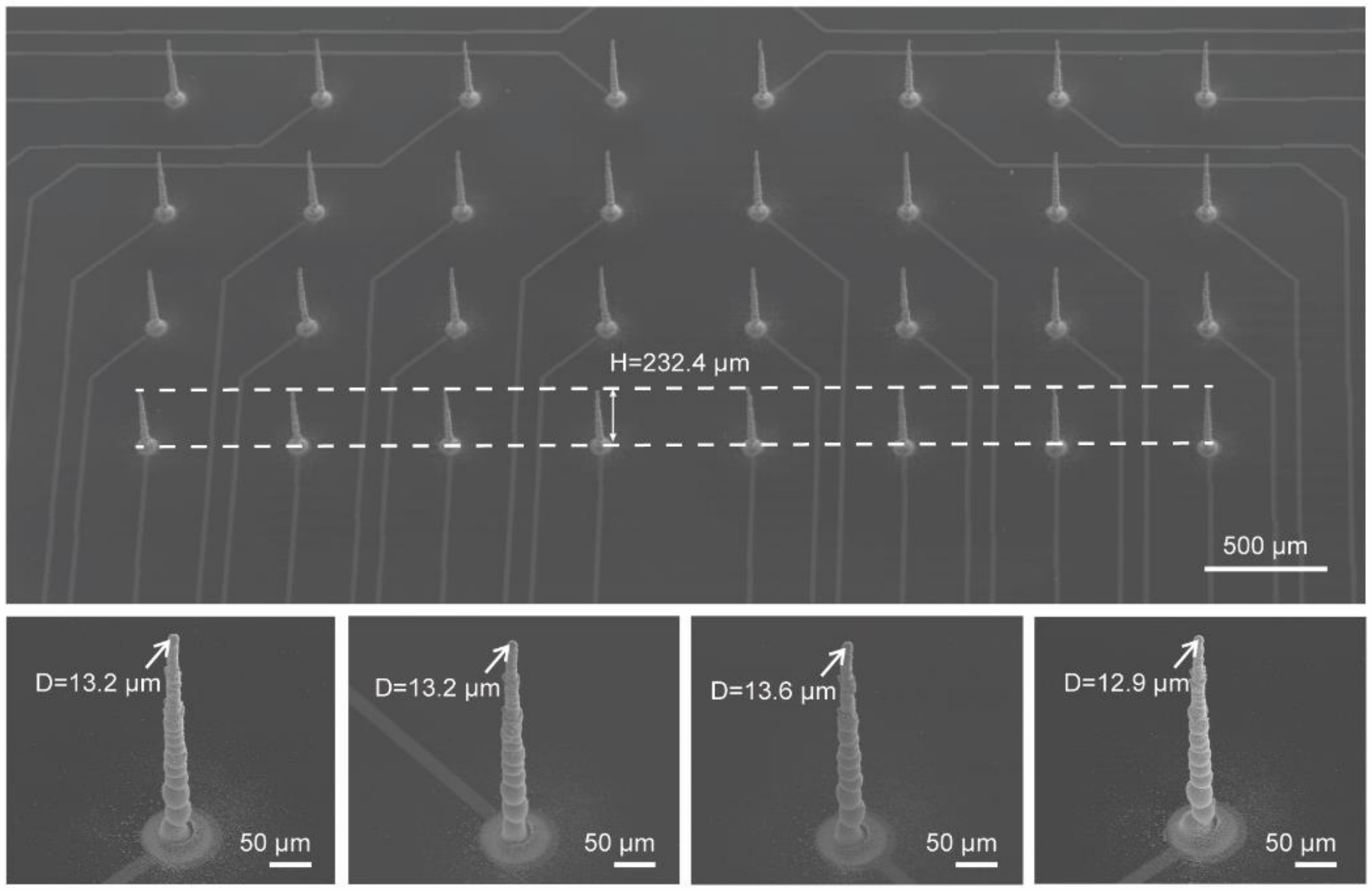

Figure S3. SEM images of the printed 32-channel microneedle array, with a magnified view showing four of the microneedles demonstrating similar heights and tip diameters.

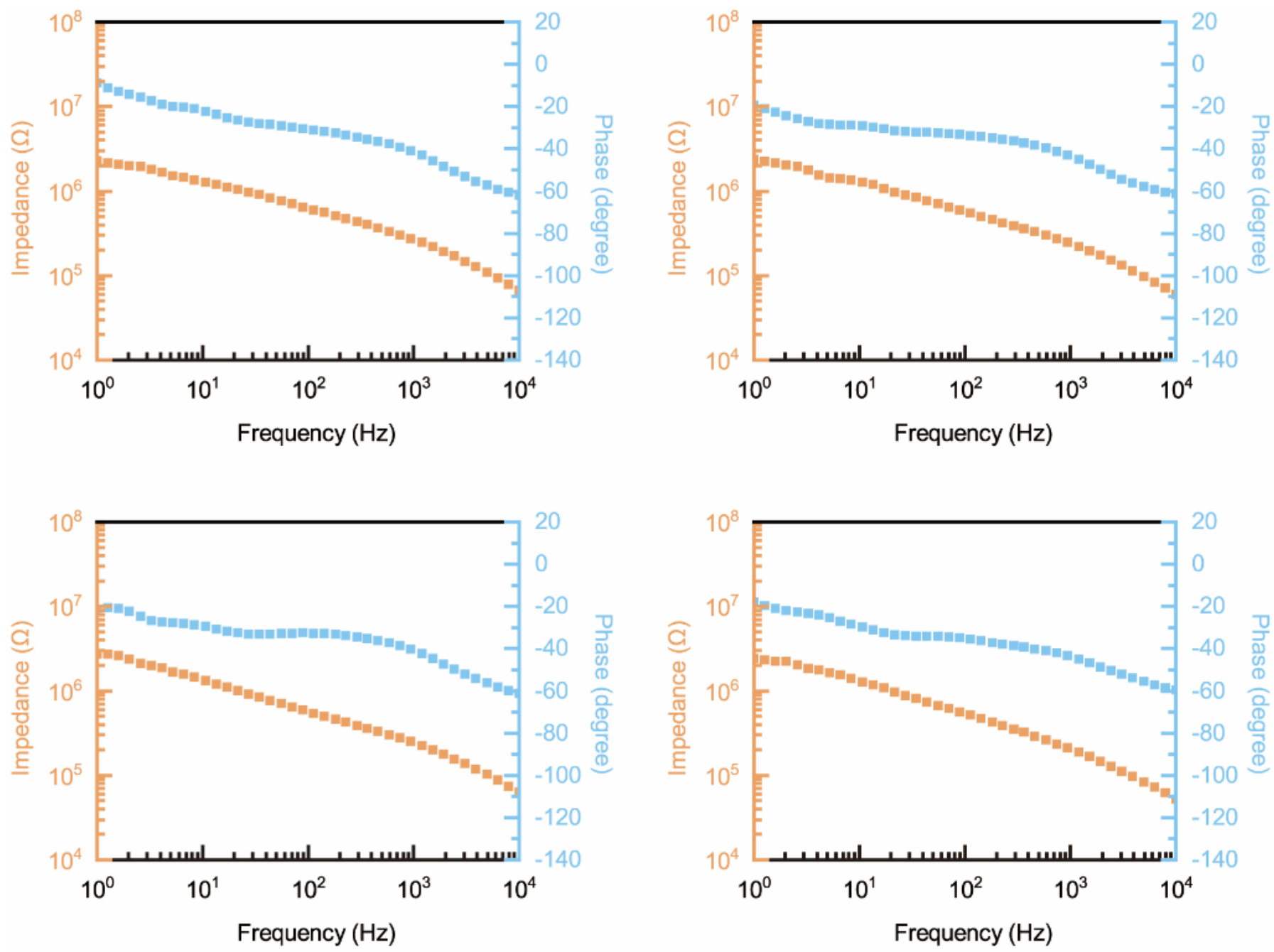

Figure S4. Electrochemical impedance spectroscopy plots of four silver microneedle electrodes.

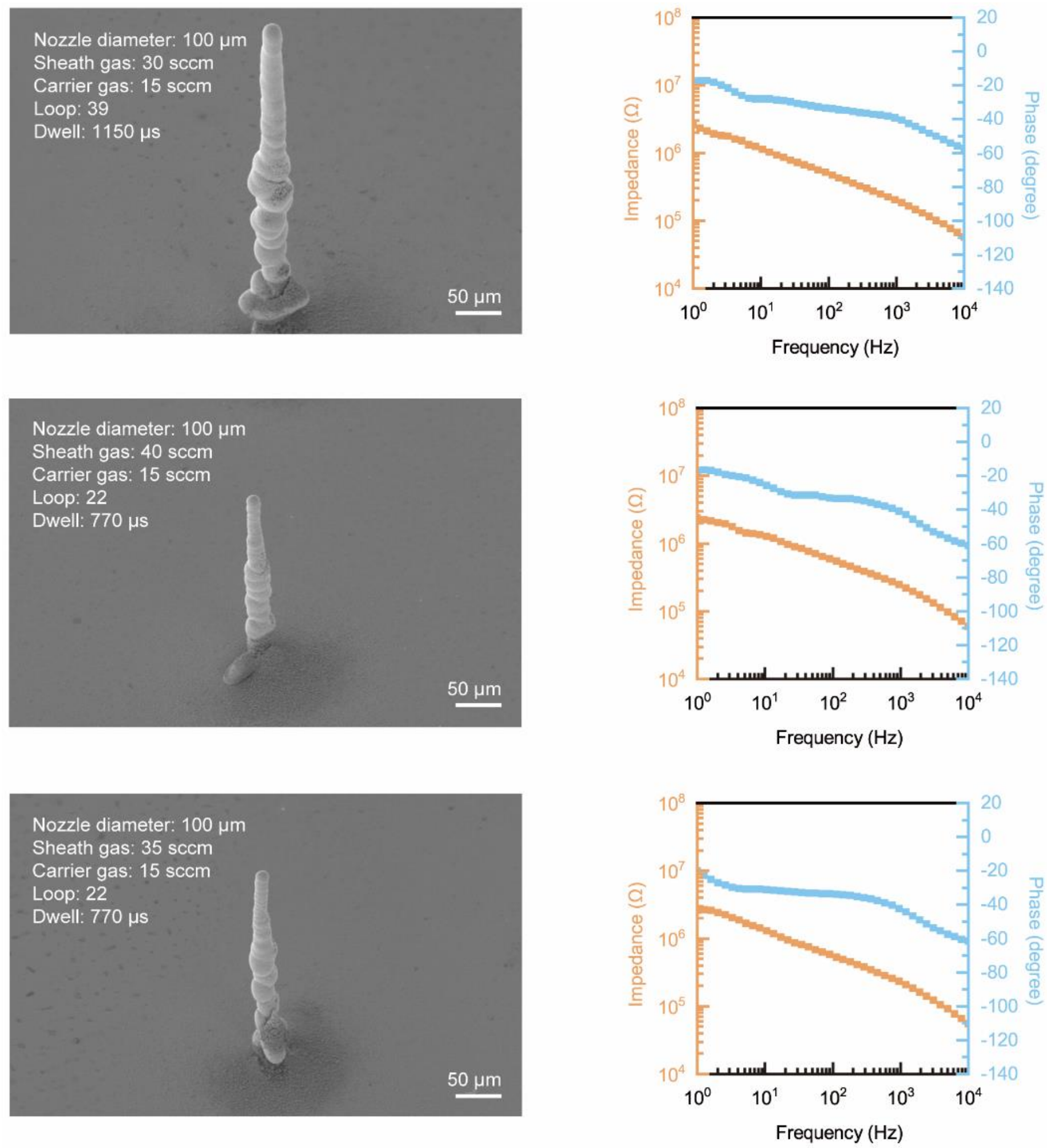

Figure S5. Printing parameters for silver microneedles and the corresponding SEM image and electrochemical impedance spectroscopy plot.

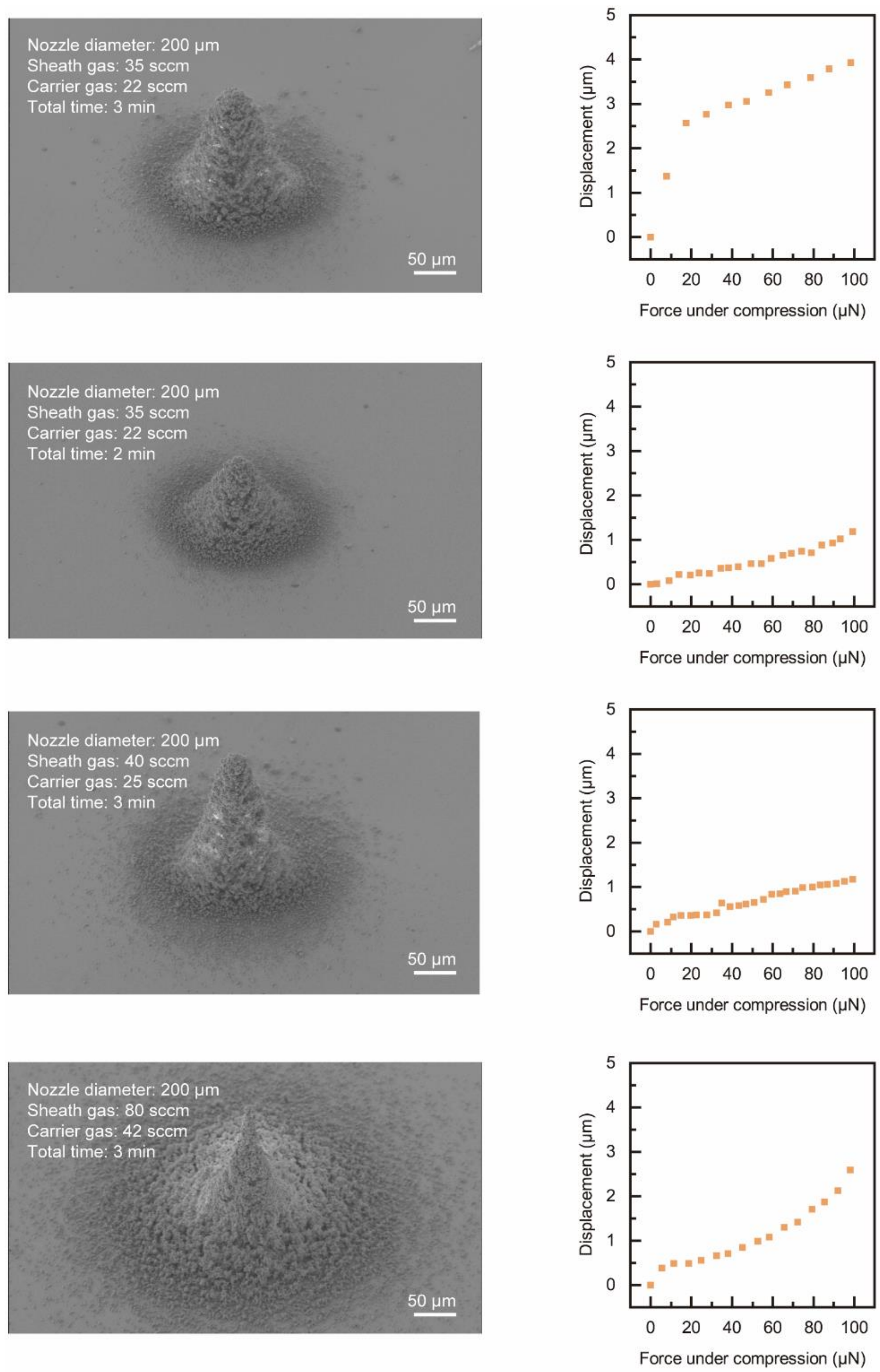

Figure S6. Printing parameters for P(VDF-TrFE) microneedles and the corresponding SEM image and force-deformation plot.

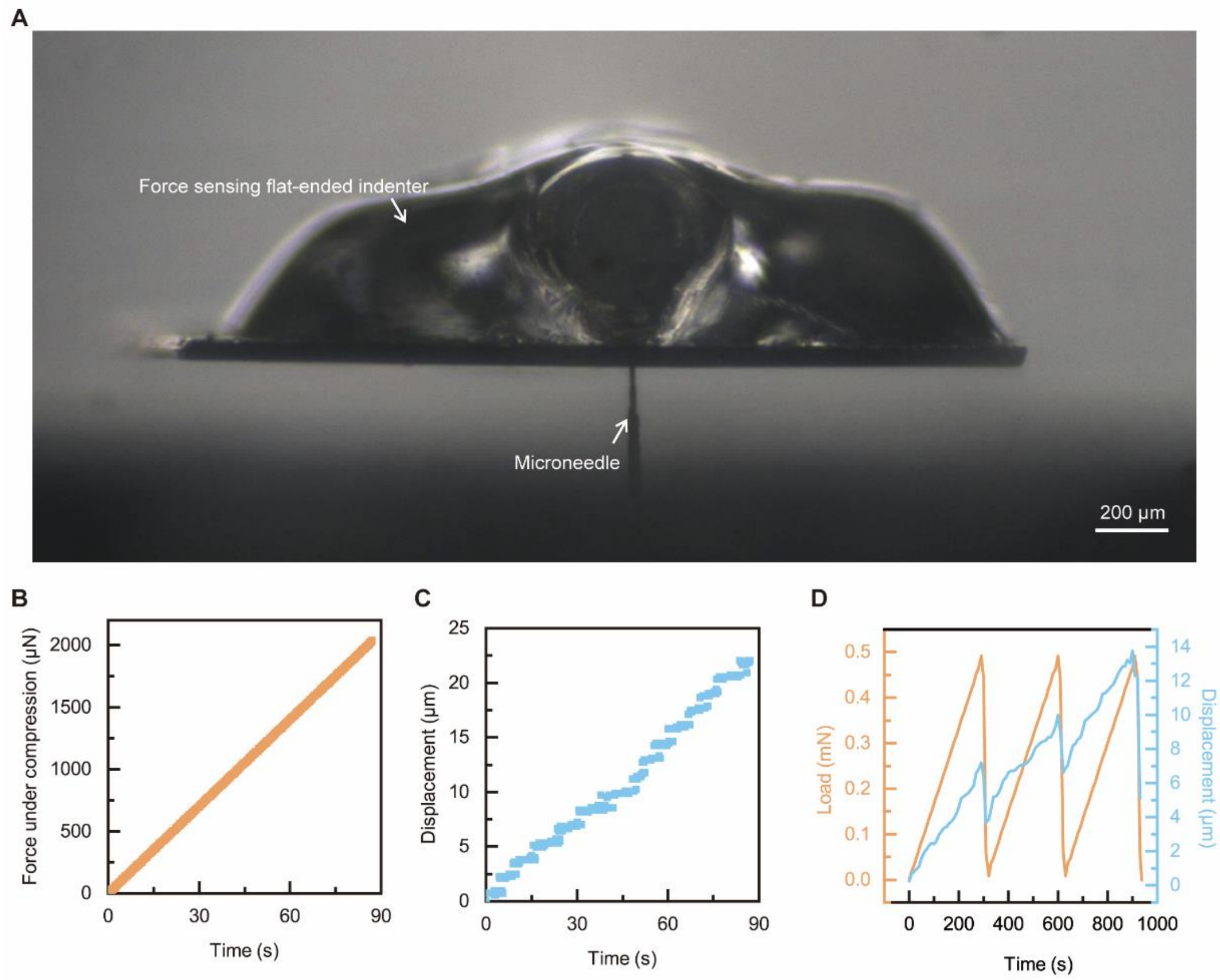

Figure S7. Mechanical strength of the silver microneedle. The image (**A**), plot of pressure versus time (**B**), and plot of tip displacement versus time (**C**) of the compression test**.** (**D**) Results of the cyclic stress test for the silver microneedle.

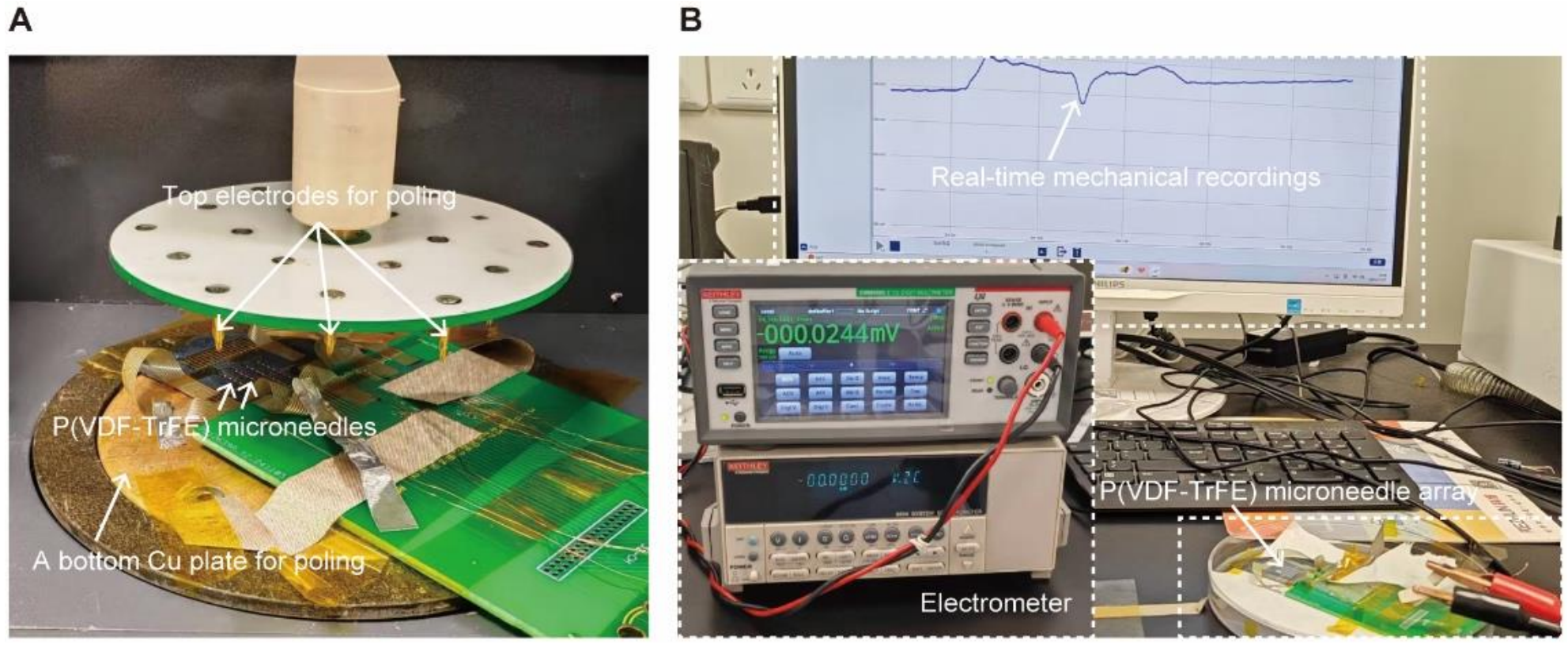

Figure S8. The experiment setup of piezoelectric signal acquisition. (**A**) Images of the high-voltage corona poling procedure. (**B**) Images of the real-time piezoelectric signal acquisition setup based on a digital multimeter.

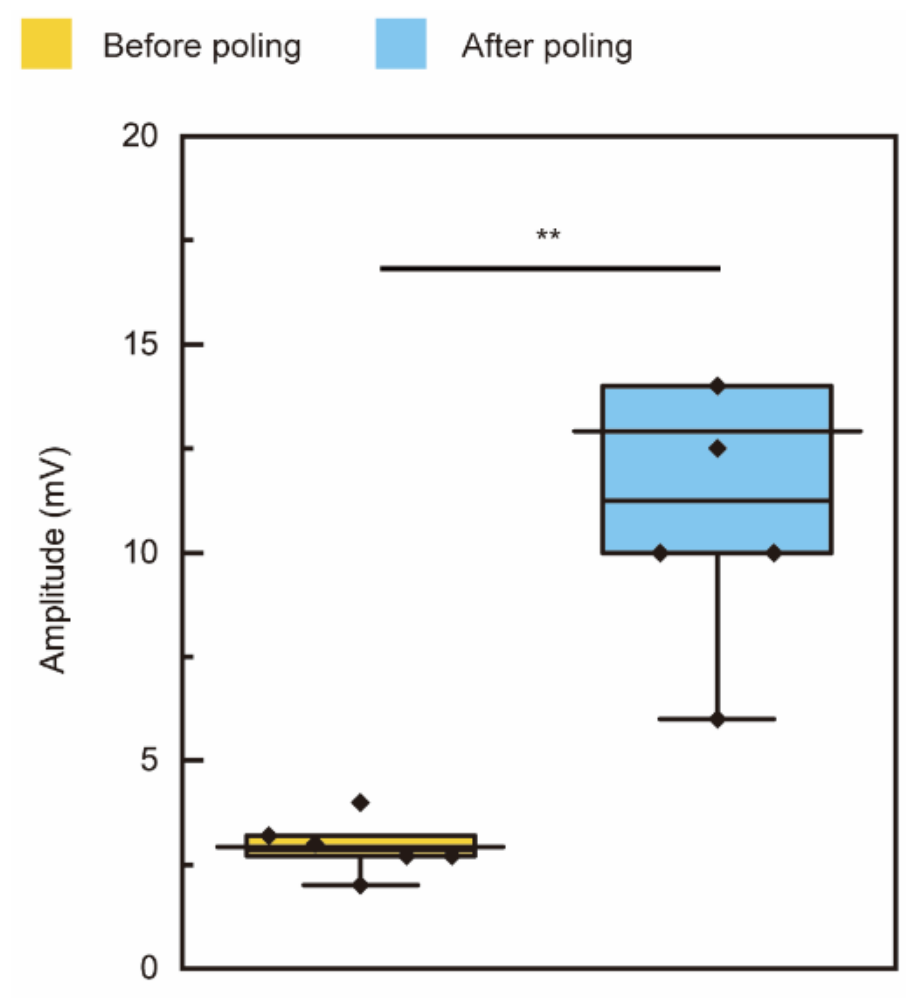

Figure S9. Increase in amplitude of the piezoelectric signal after the poling treatment.

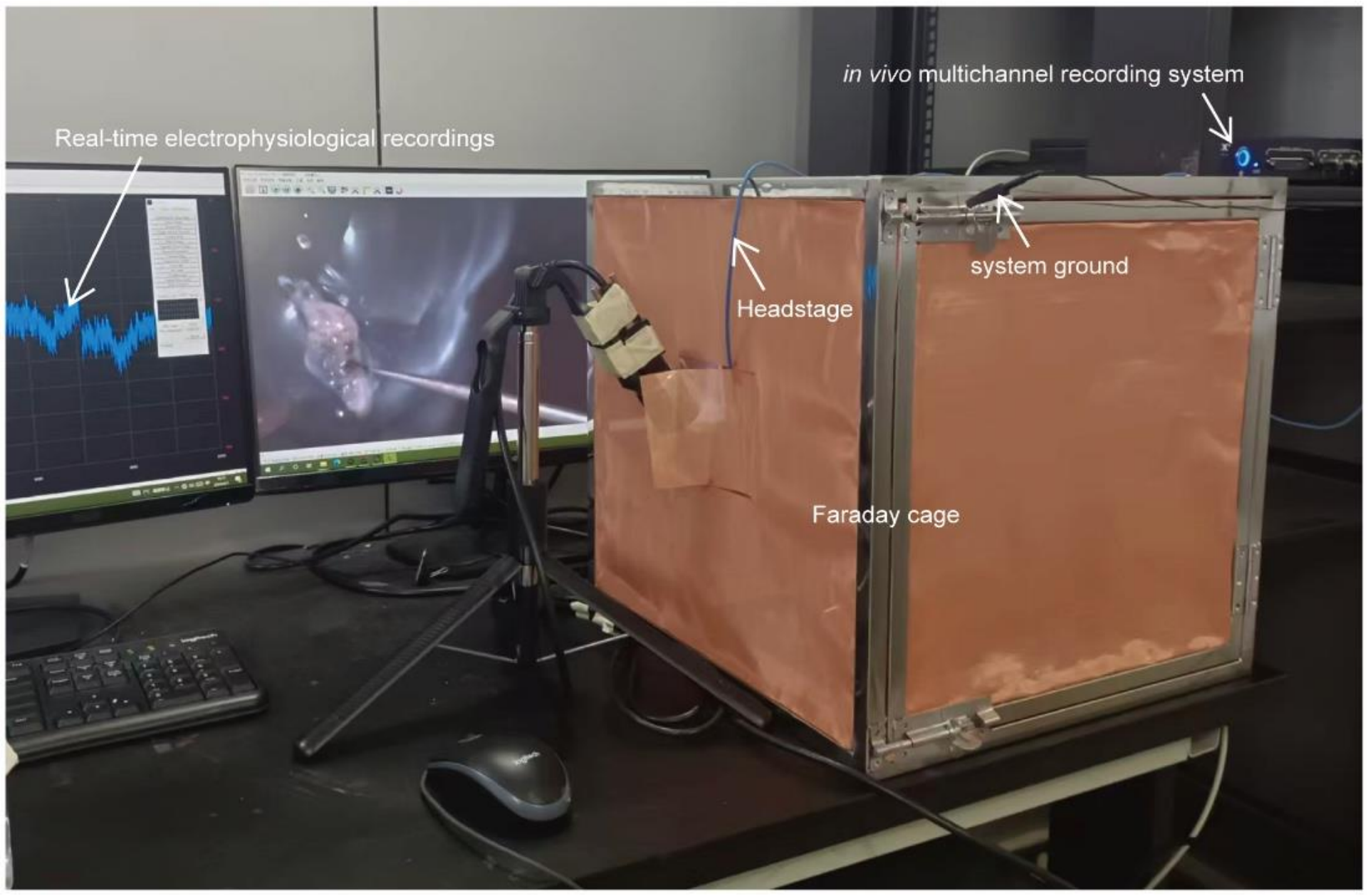

Figure S10. A Faraday cage-based electrophysiological recording setup.

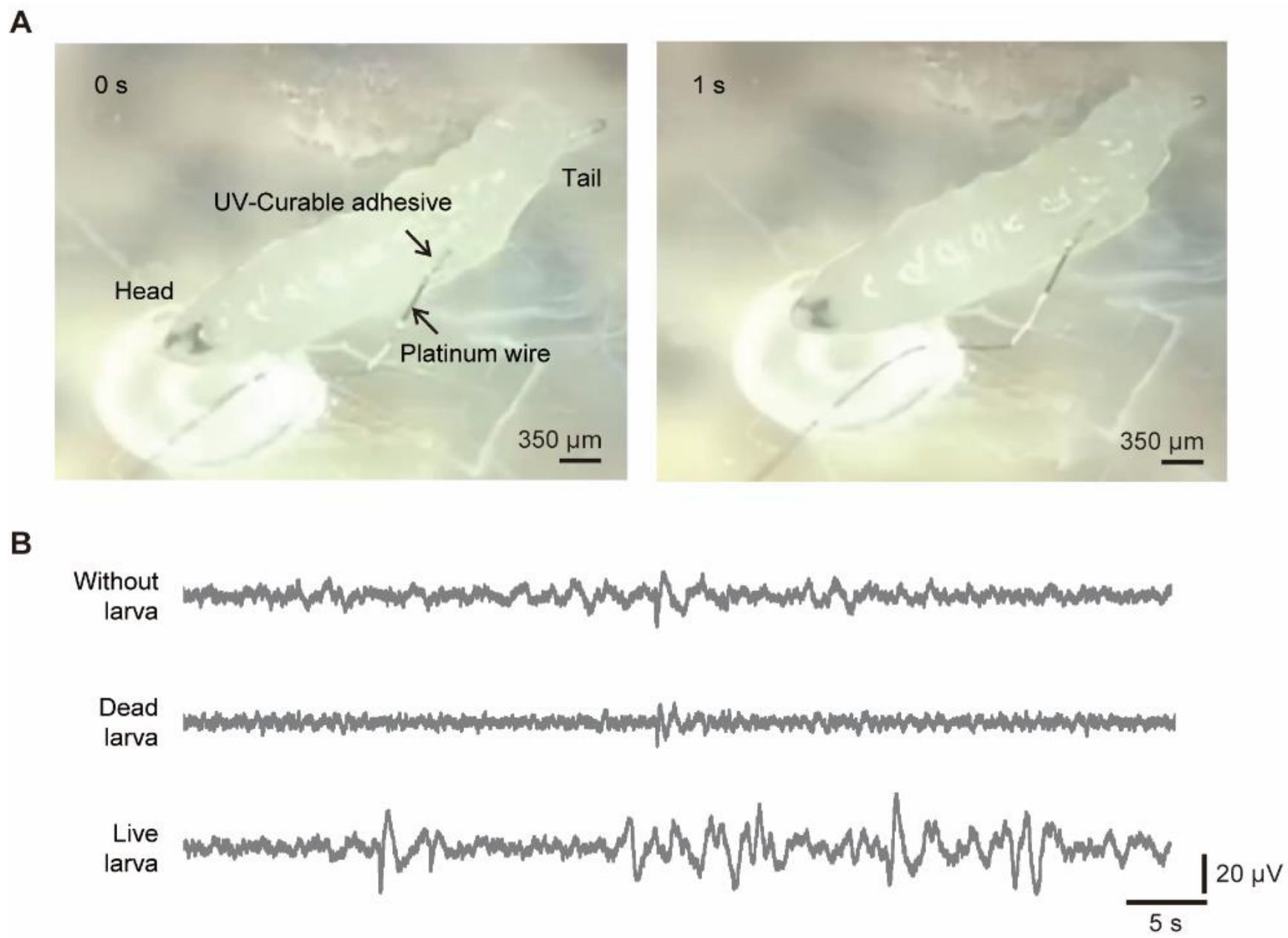

Figure S11. Electrophysiology recording of an immobilized larva with a platinum wire electrode. (**A**) Images of a larva with a platinum wire electrode fixed on its body. (**B**) Traces recorded from the platinum wire electrode (1 - 100 Hz band-pass filter, Butterworth, 4th order).

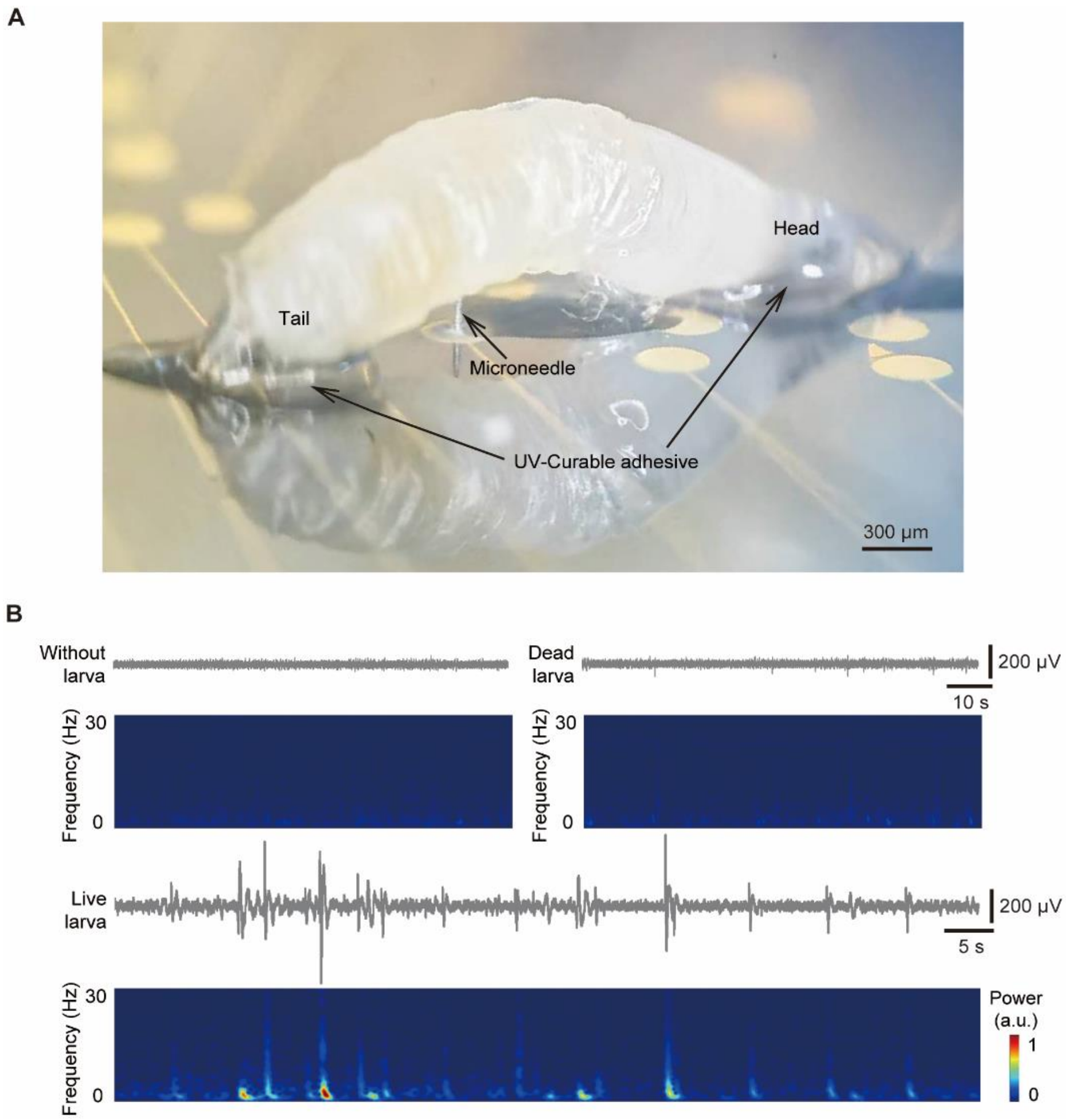

Figure S12. Electrophysiological recording of an immobilized larva with a microneedle electrode. (**A**) An image of a larva immobilized on a microneedle electrode. (**B**) Representative traces and spectrograms recorded from the microneedle electrode (1–100 Hz band-pass filter, Butterworth, 4th order) under three conditions: no larvae, dead larvae, and live larvae.

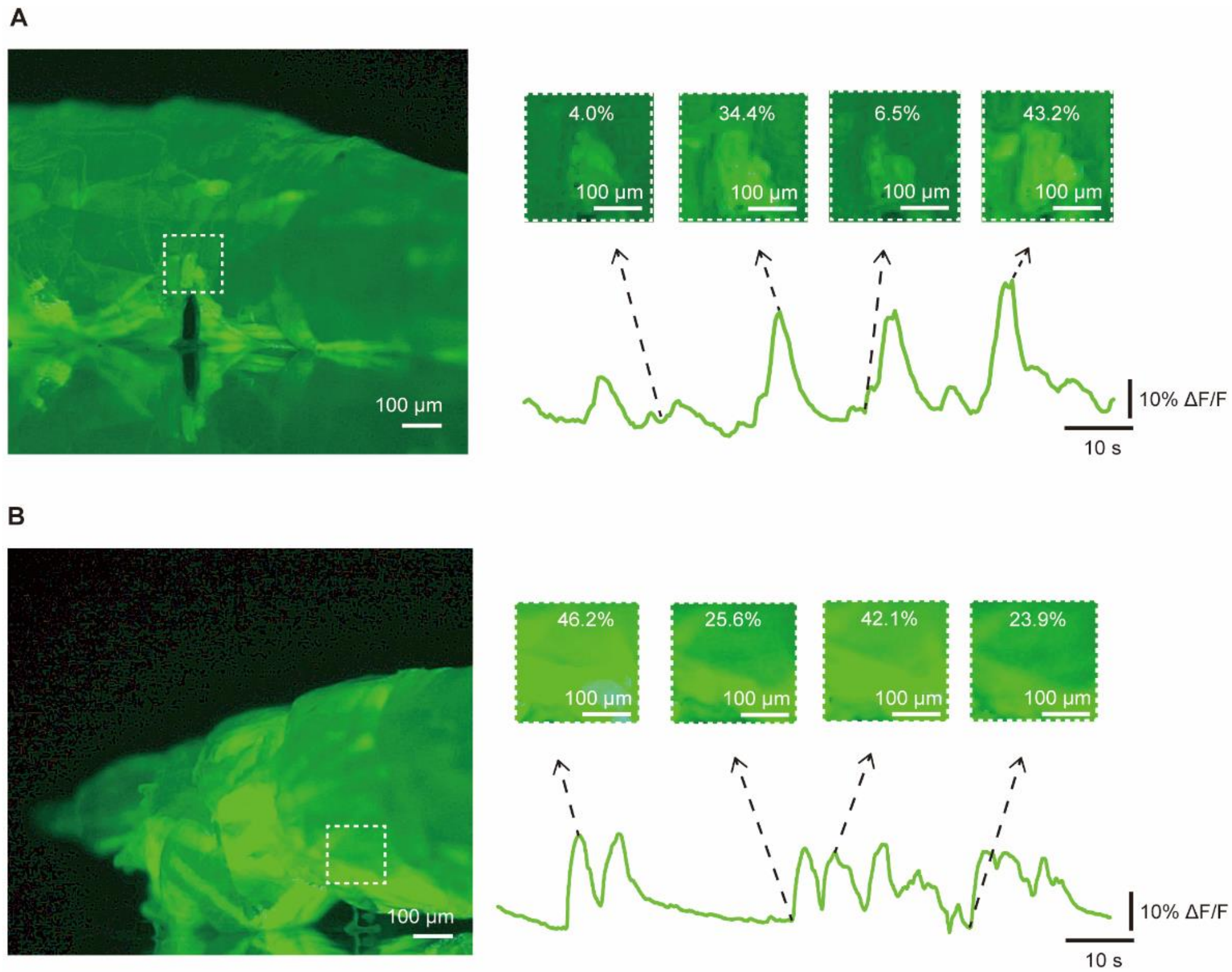

Figure S13. Calcium imaging of the electrode-larva interface. Contact state between the microneedle and the immobilized larva, with segments of abdominal (**A**) and tail (**B**) under either contraction or relaxation.

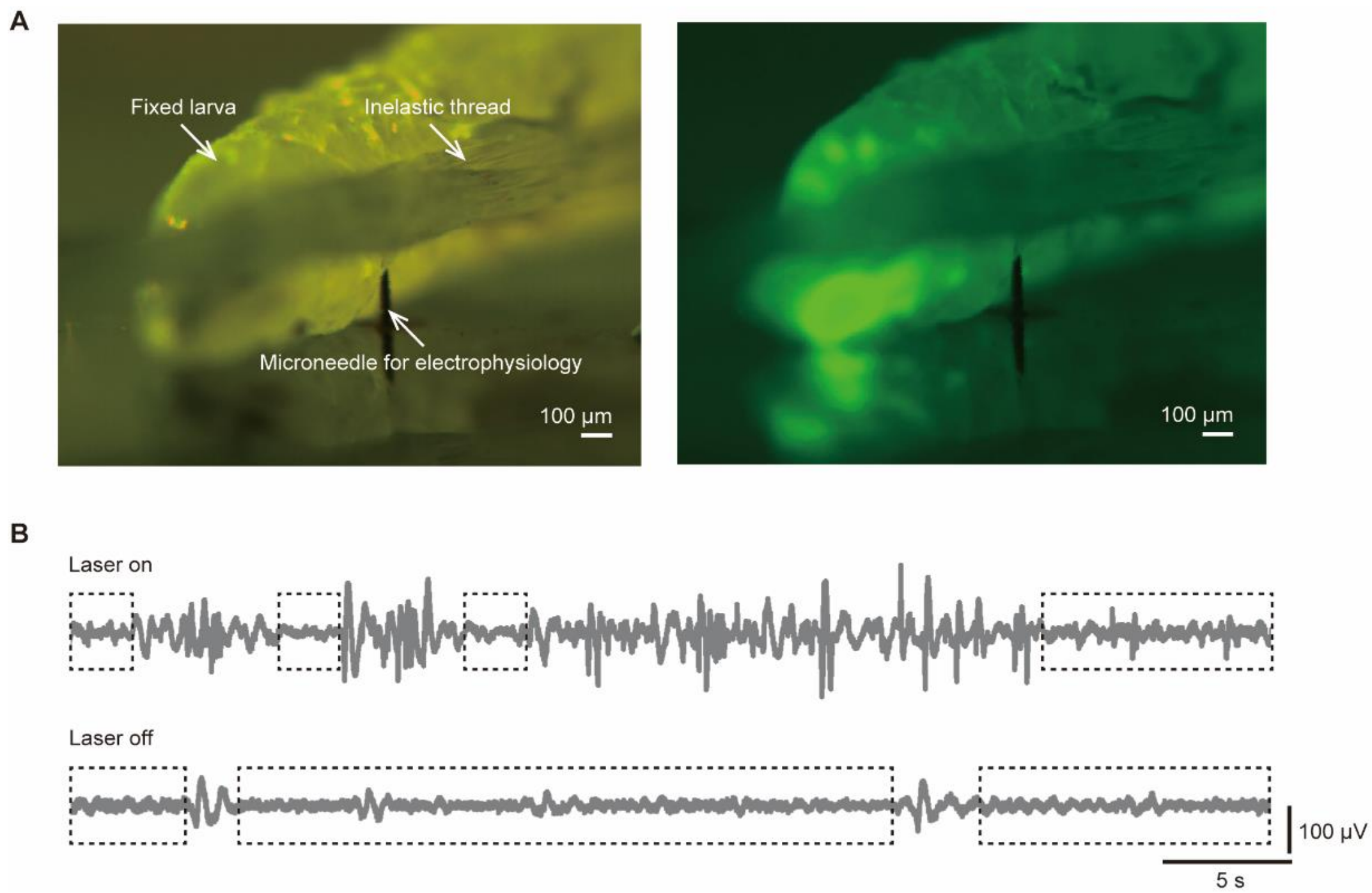

Figure S14. Evaluation of the noise baseline in electrophysiological recordings during laser-on and laser-off periods. (**A**) Images of the experiment setup. (**B**) Traces recorded from the microneedle electrode (1 - 100 Hz band-pass filter, Butterworth, 4th order).

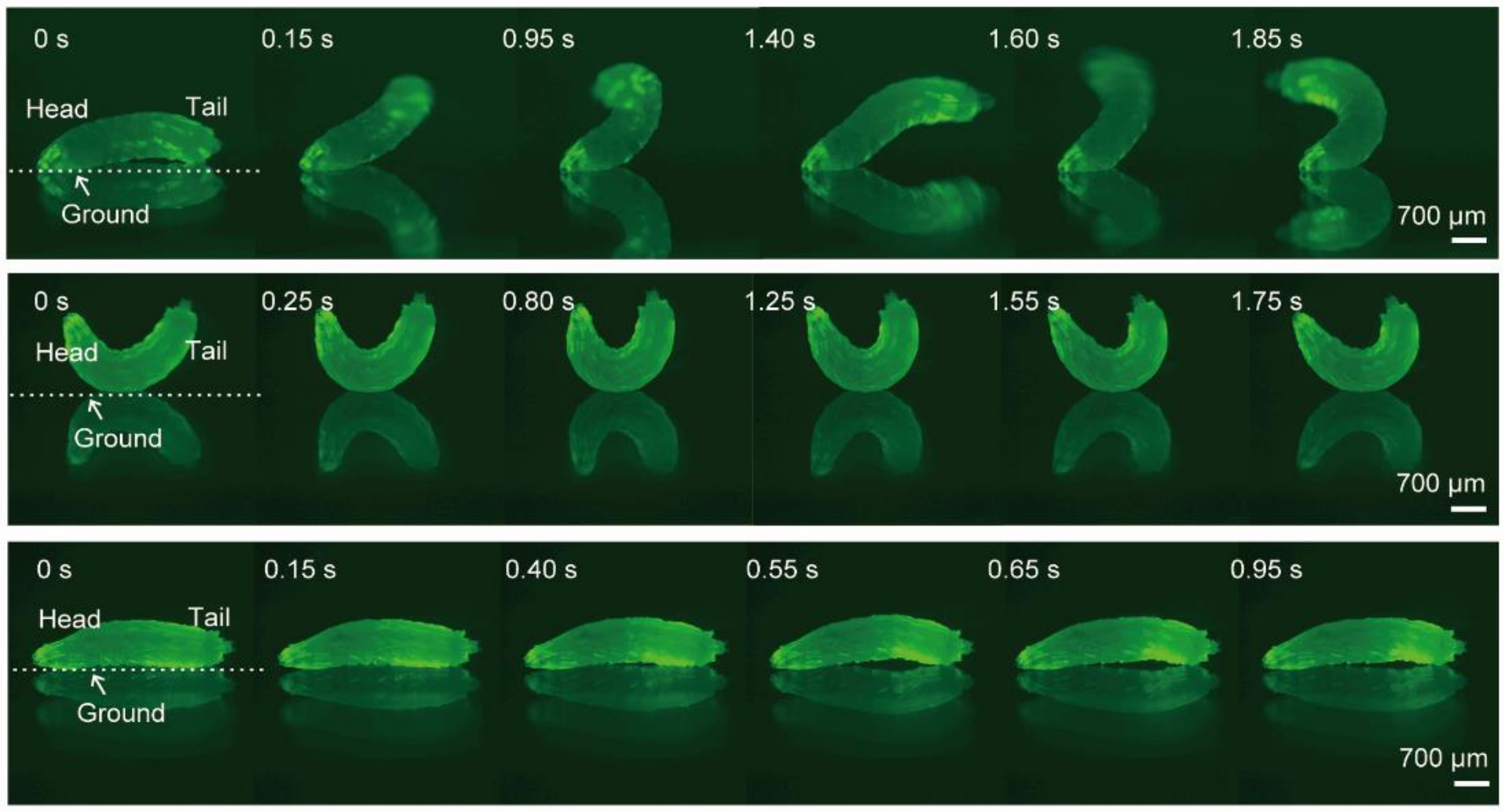

Figure S15. Calcium imaging sequence capturing 3D movements of a freely moving *Drosophila* larva.

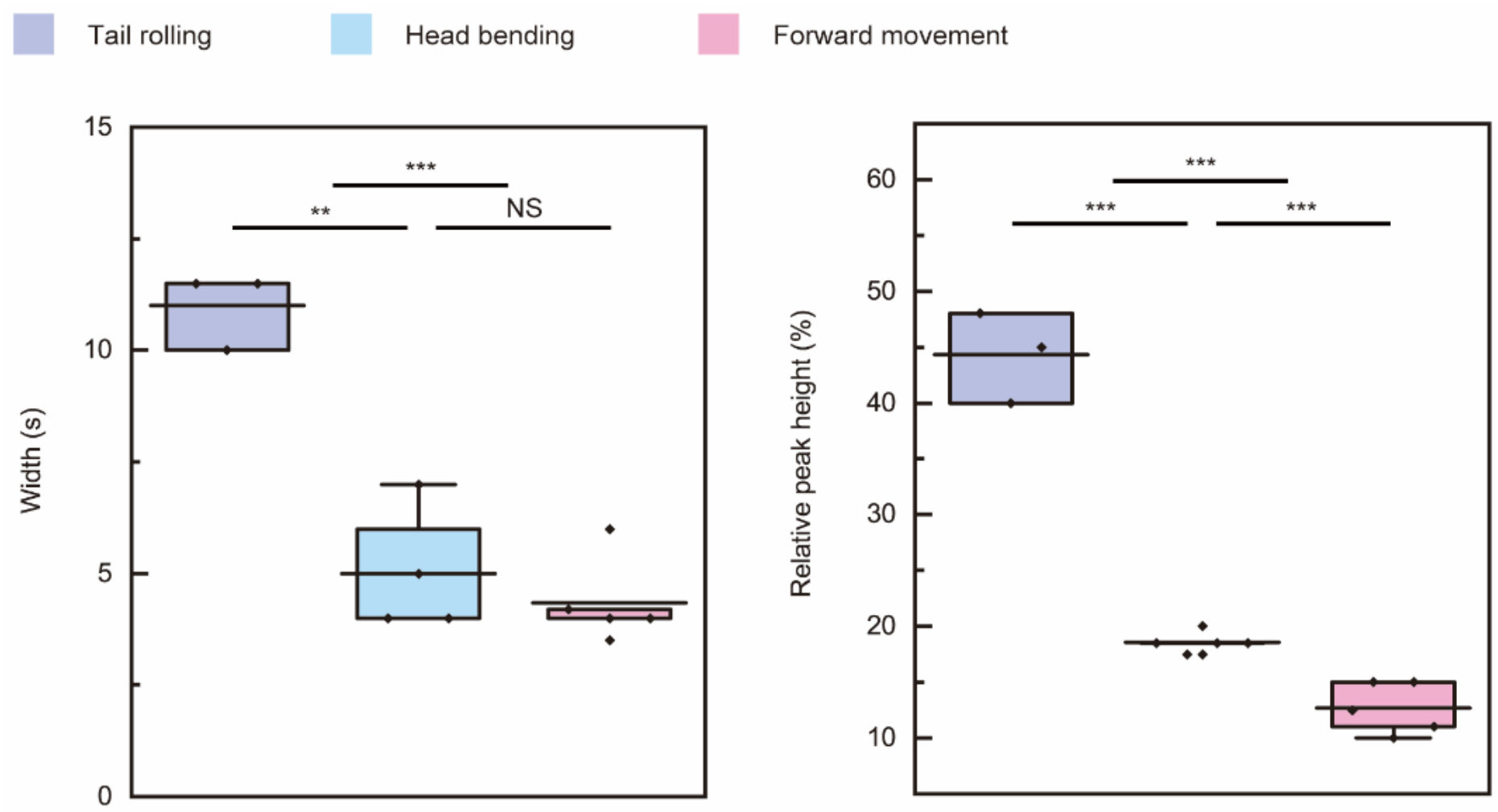

Figure S16. Features of the mechanical signals recorded from a *Drosophila* larva with different 3D movements.

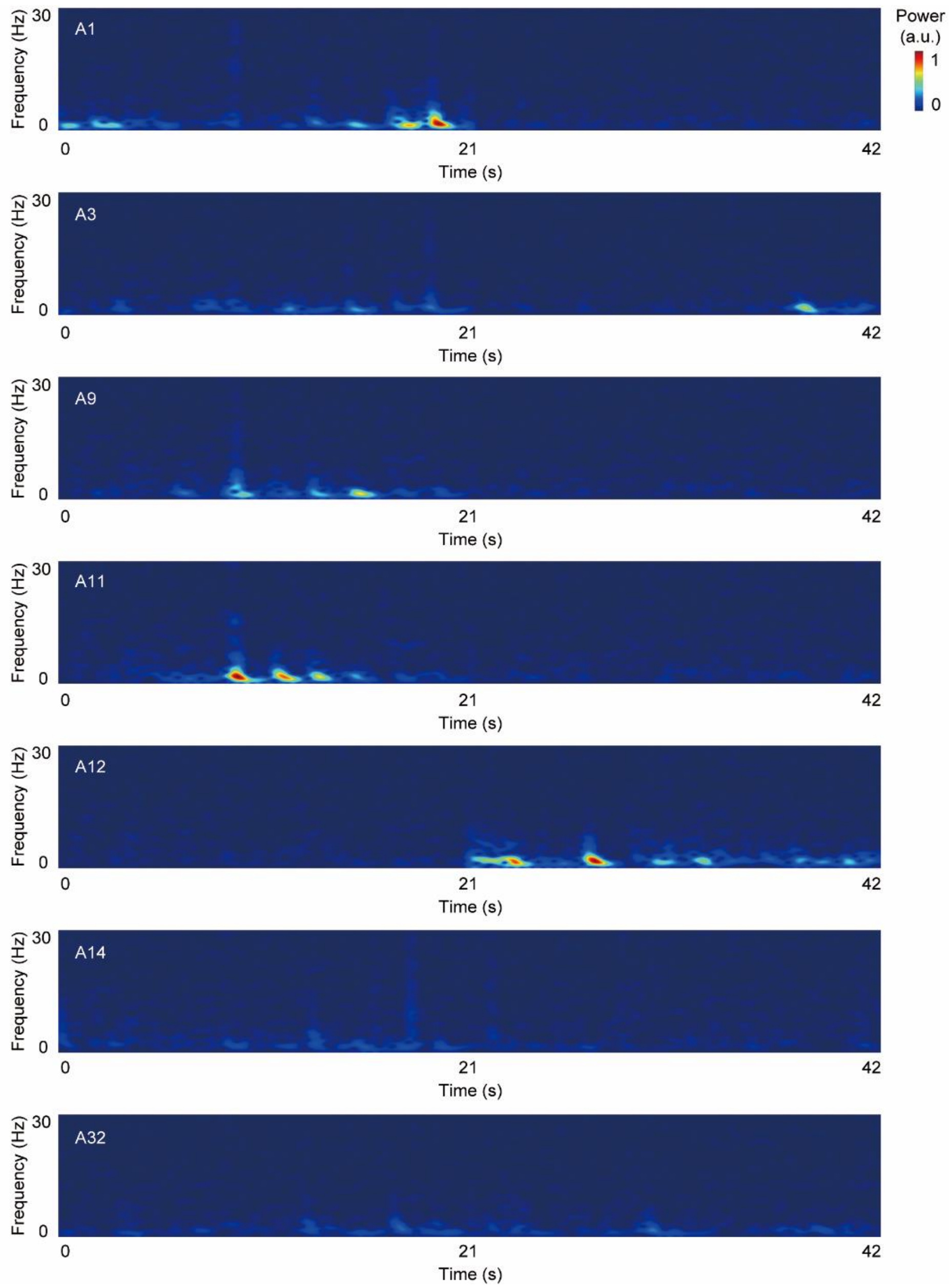

Figure S17. Normalized spectrograms of electrophysiological recordings, corresponding to the channels in Figure 4B, show the dramatic changes in the amplitude and frequency when the larva is freely moving.

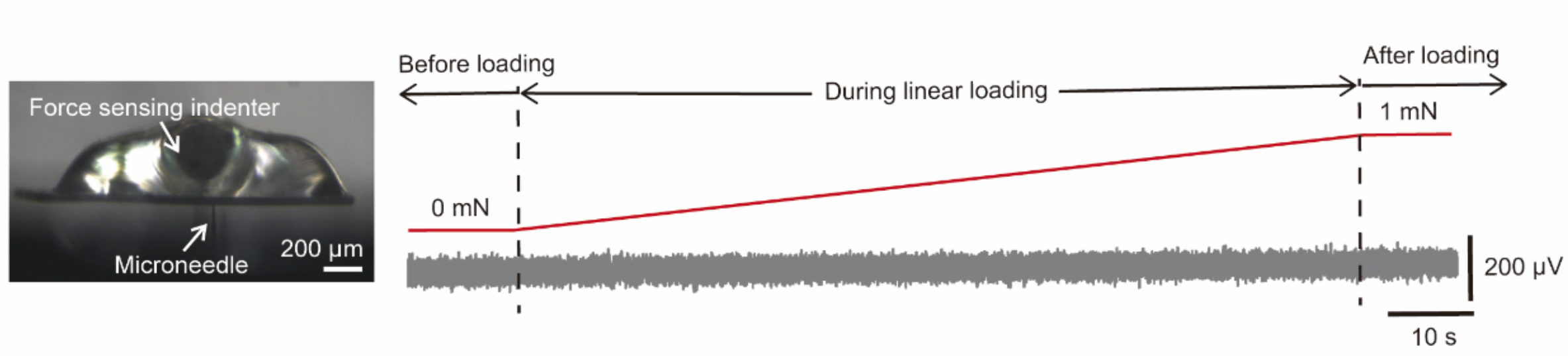

Figure S18. The recorded electrical signal during mechanical loading.

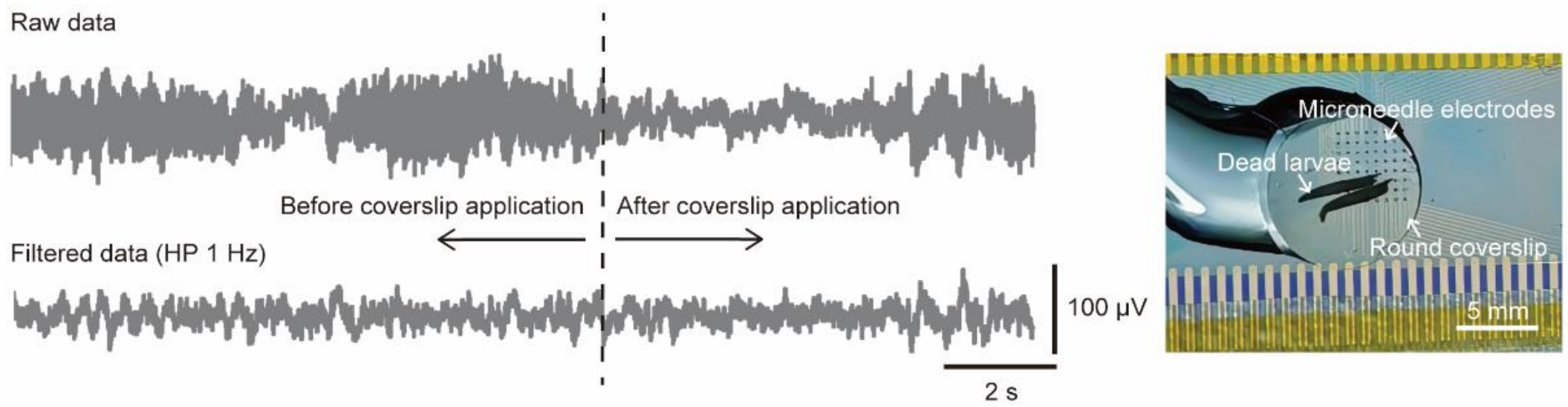

Figure S19. Electrical signal recorded by a silver microneedle electrode from the dead larva subjected to externally induced displacement.

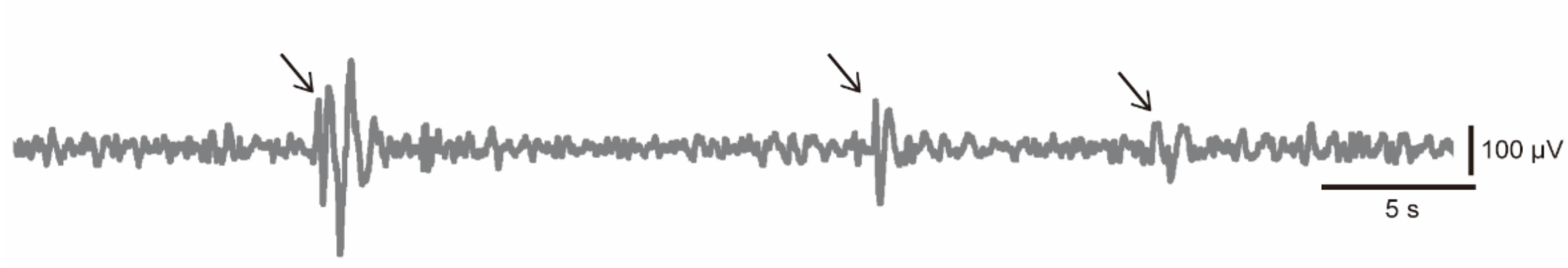

Figure S20. Electrophysiological signals of a live larva with minimizing mechanical deformation.

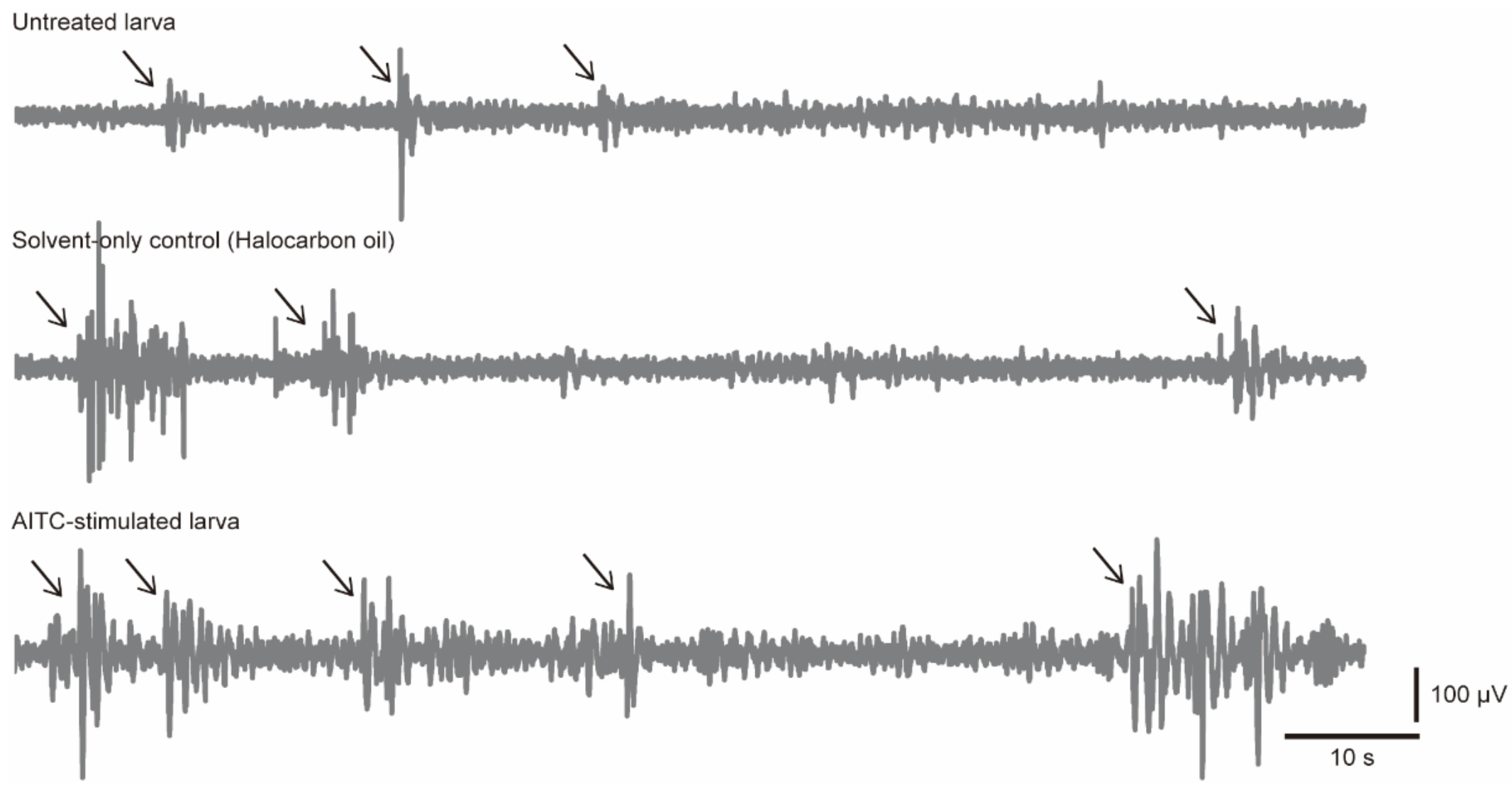

Figure S21. Electrophysiological recordings of freely behaving larva with untreated, Halocarbon oil solvent, and Allyl Isothiocyanate (AITC) treatments.

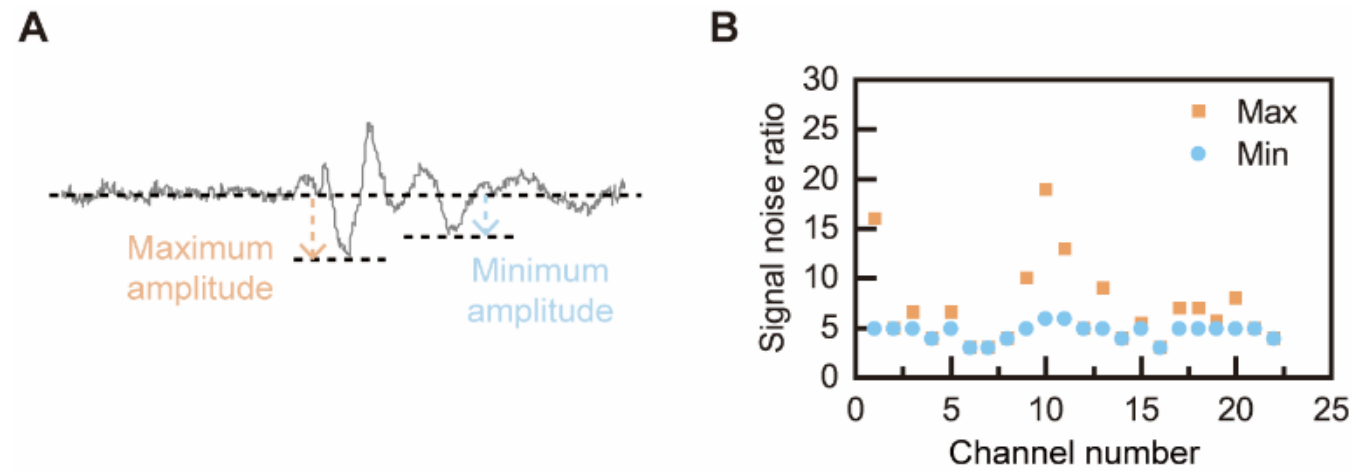

Figure S22. Signal quality assessment of the electrophysiological recordings. (**A**) Illustration of the waveform features. (**B**) Statistical results of the SNR across electrophysiological channels.

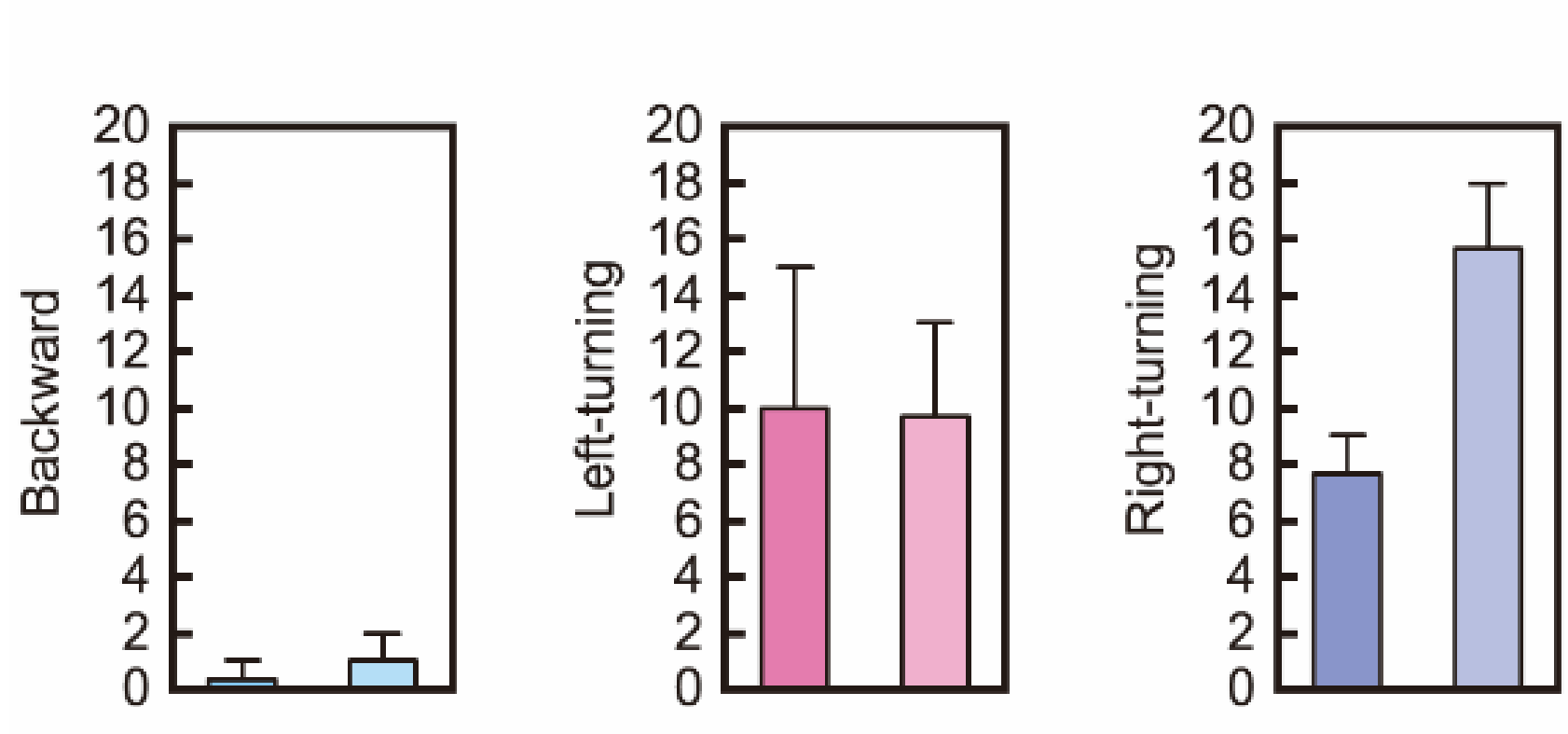

Figure S23. Behavior category statistics of a 10-minute free-moving activity of a larva in the experimental environment with and without microneedles (n=3).

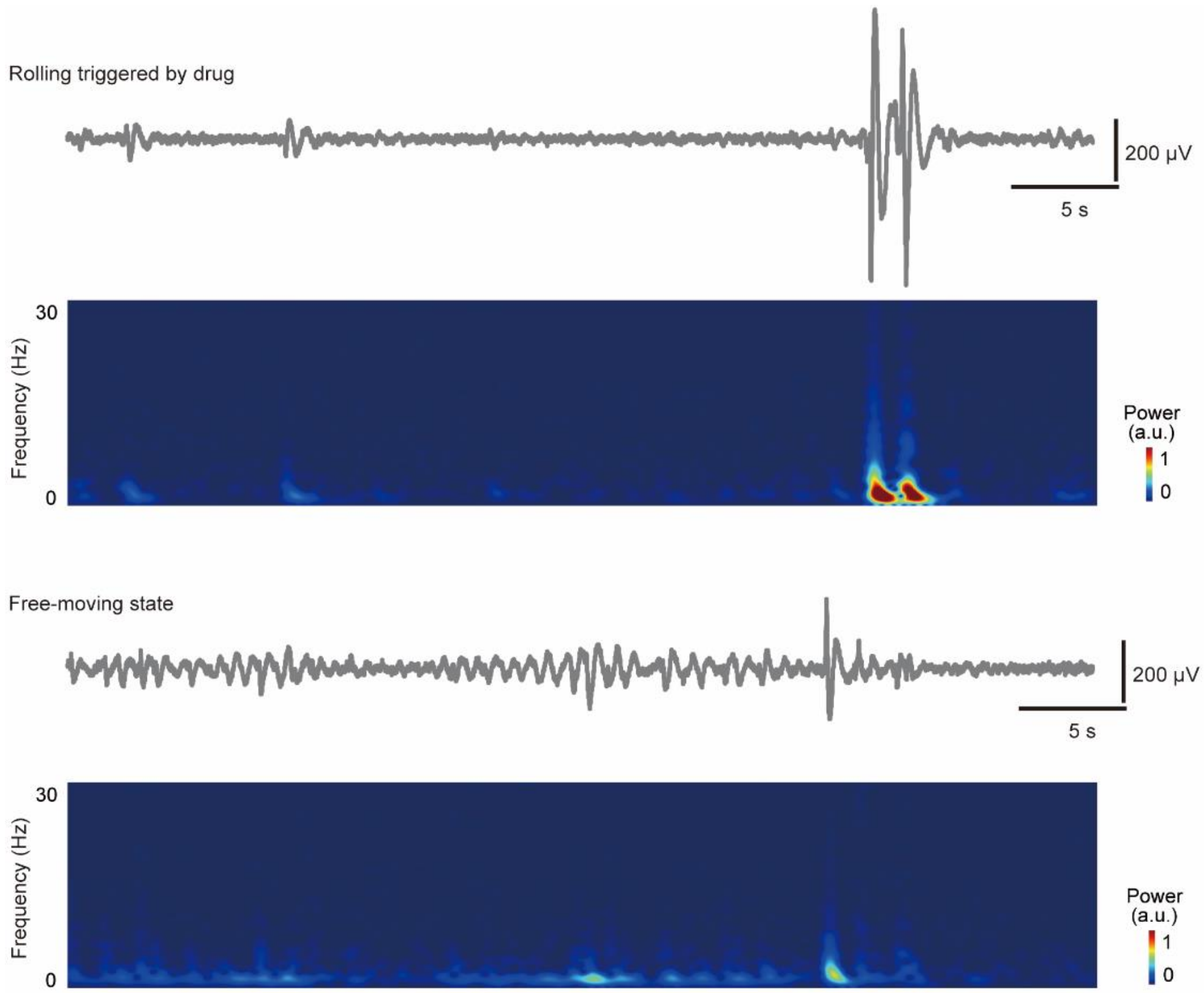

Figure S24. Comparison of electrophysiological signals of rolling escape behavior under drug stimulation versus those under normal free-moving recording conditions.

$$☿\ \frac{Mef2\text{-}GAL4}{Mef2\text{-}GAL4} \times ♂\ \frac{TRiP.HMS00868}{TRiP.HMS00868}\ (UAS\text{-}para\text{-}RNAi)$$

$$☿\ \frac{Mef2\text{-}GAL4}{Mef2\text{-}GAL4} \times ♂\ \frac{TRiP.JF02146}{TRiP.JF02146}\ (UAS\text{-}slo\text{-}RNAi)$$

Figure S25. Genetic strategy for tissue-specific RNAi-mediated knockdown of *para* and *slo*.

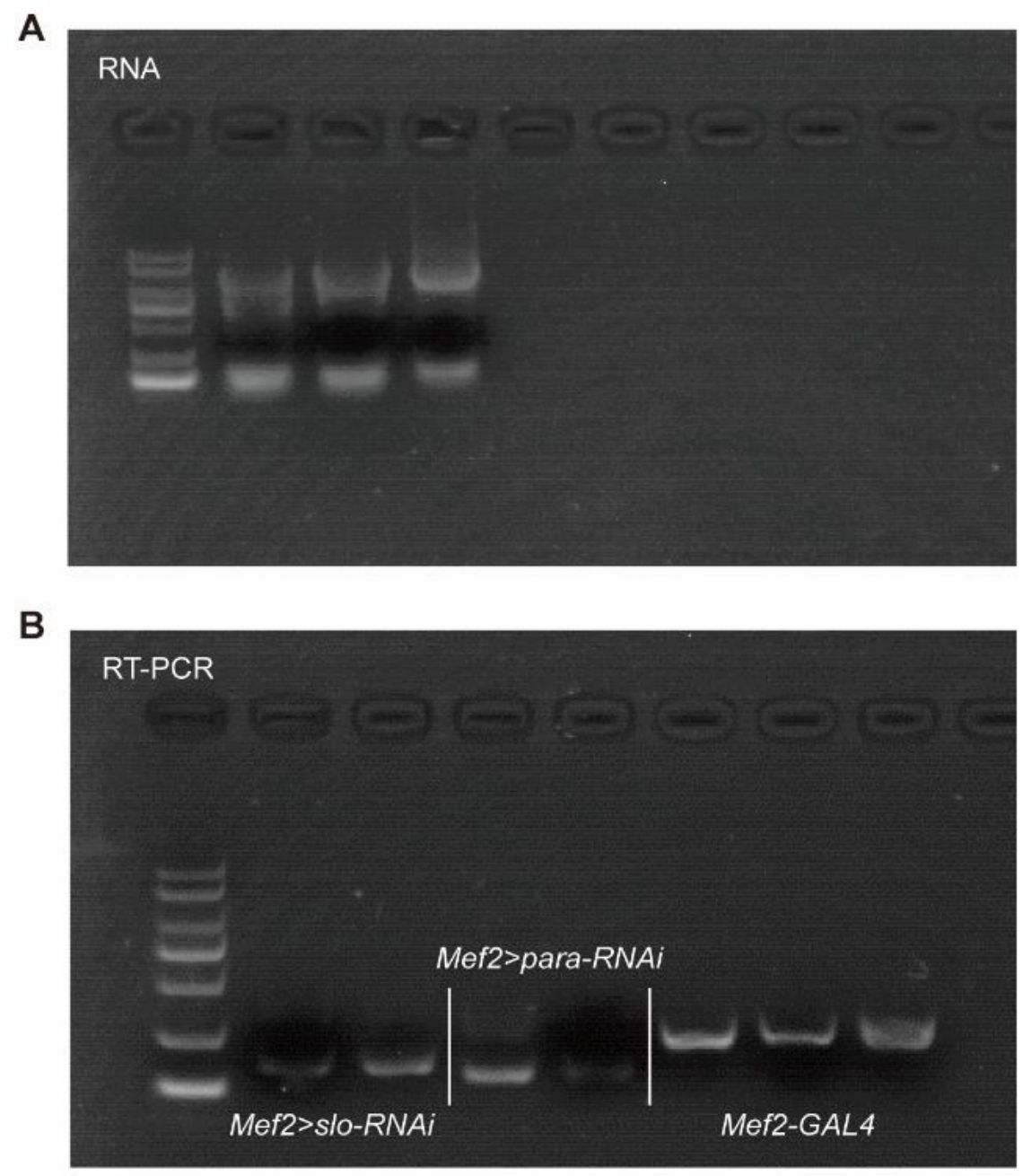

Figure S26. Quality control of RNA samples and primer validation. Agarose gel electrophoresis of total RNA (**A**) and conventional RT-PCR products (**B**) for primer validation before quantitative PCR (qPCR). The gel images confirm RNA integrity and primer specificity.

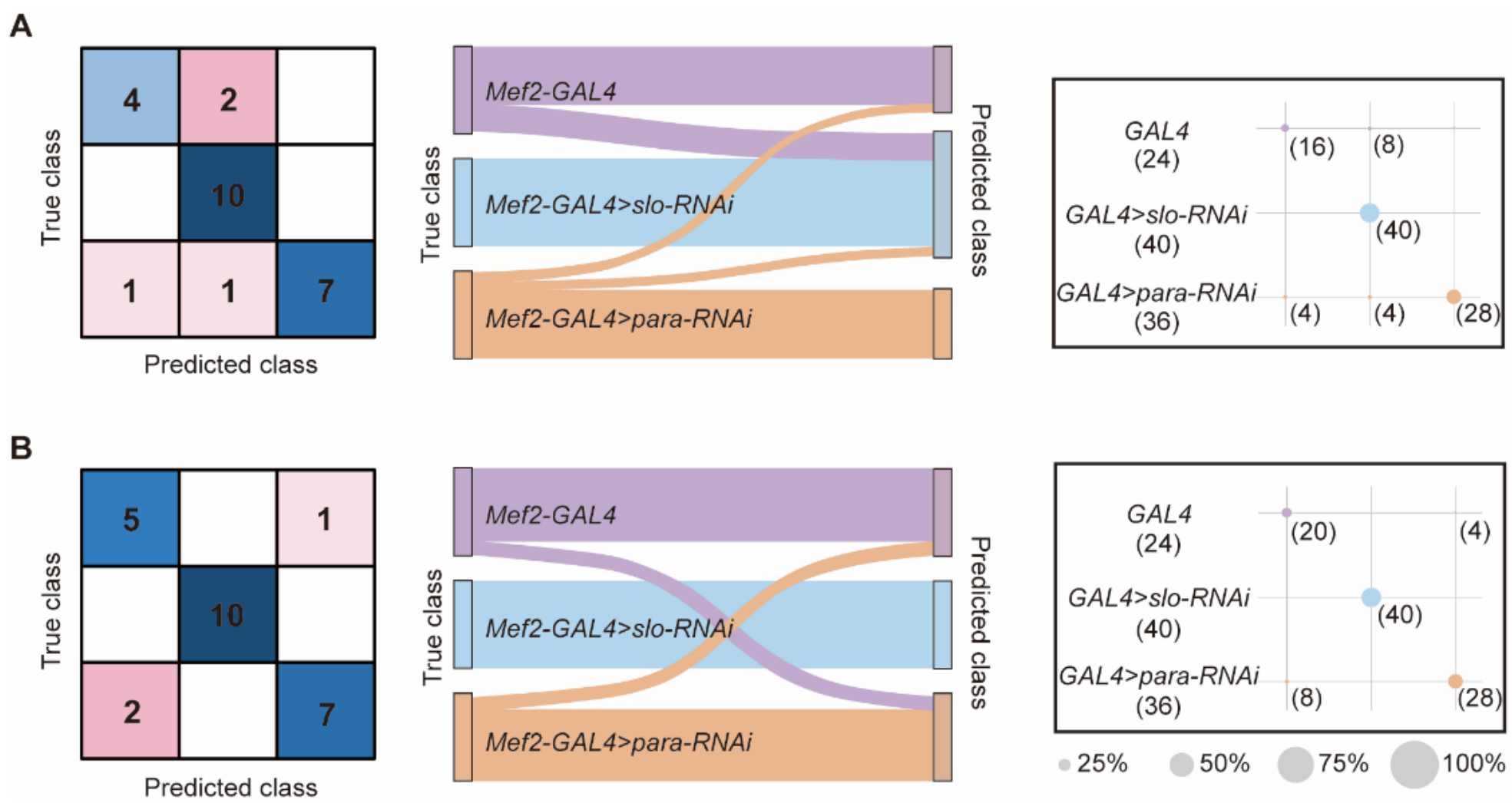

Figure S27. Results of the cross-modal genotype mapping (CMGM) with a machine-learning model on the recorded electrophysiological waveforms. (**A**) The CMGM results, derived from two quantified features, were visualized and analyzed using a confusion matrix, a river plot, and a dot plot. (**B**) The CMGM results, derived from four quantified features, were visualized and analyzed in the same way as in (**A**).

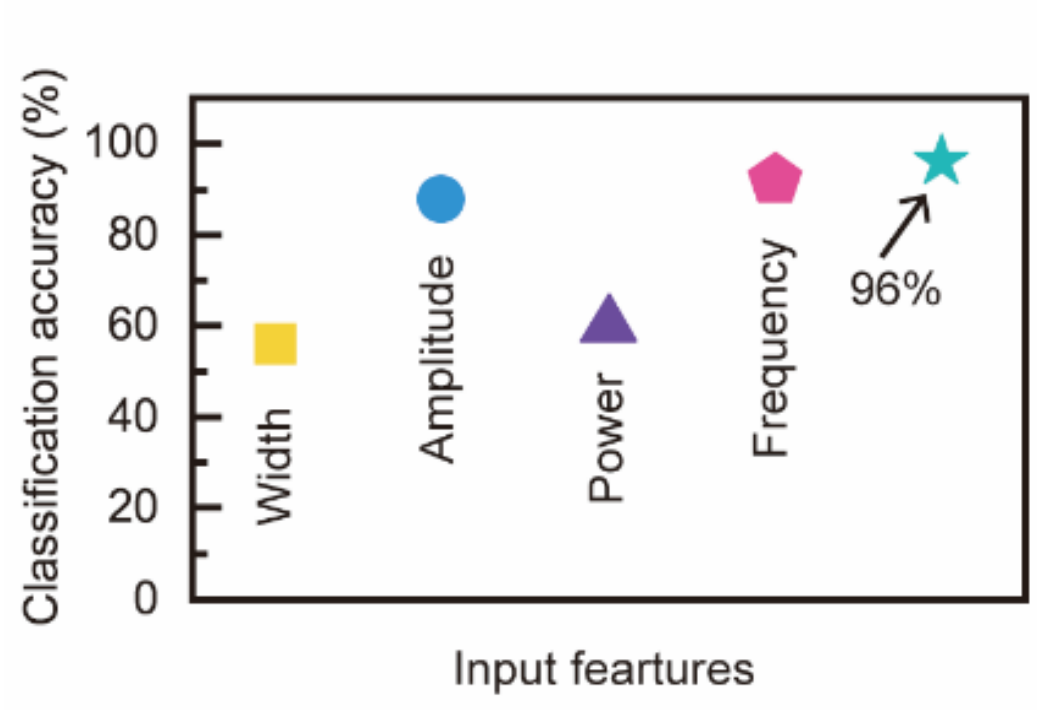

Figure S28. Accuracy of genotype classification using electrophysiological features alone versus using combined electrophysiological and mechanical features.

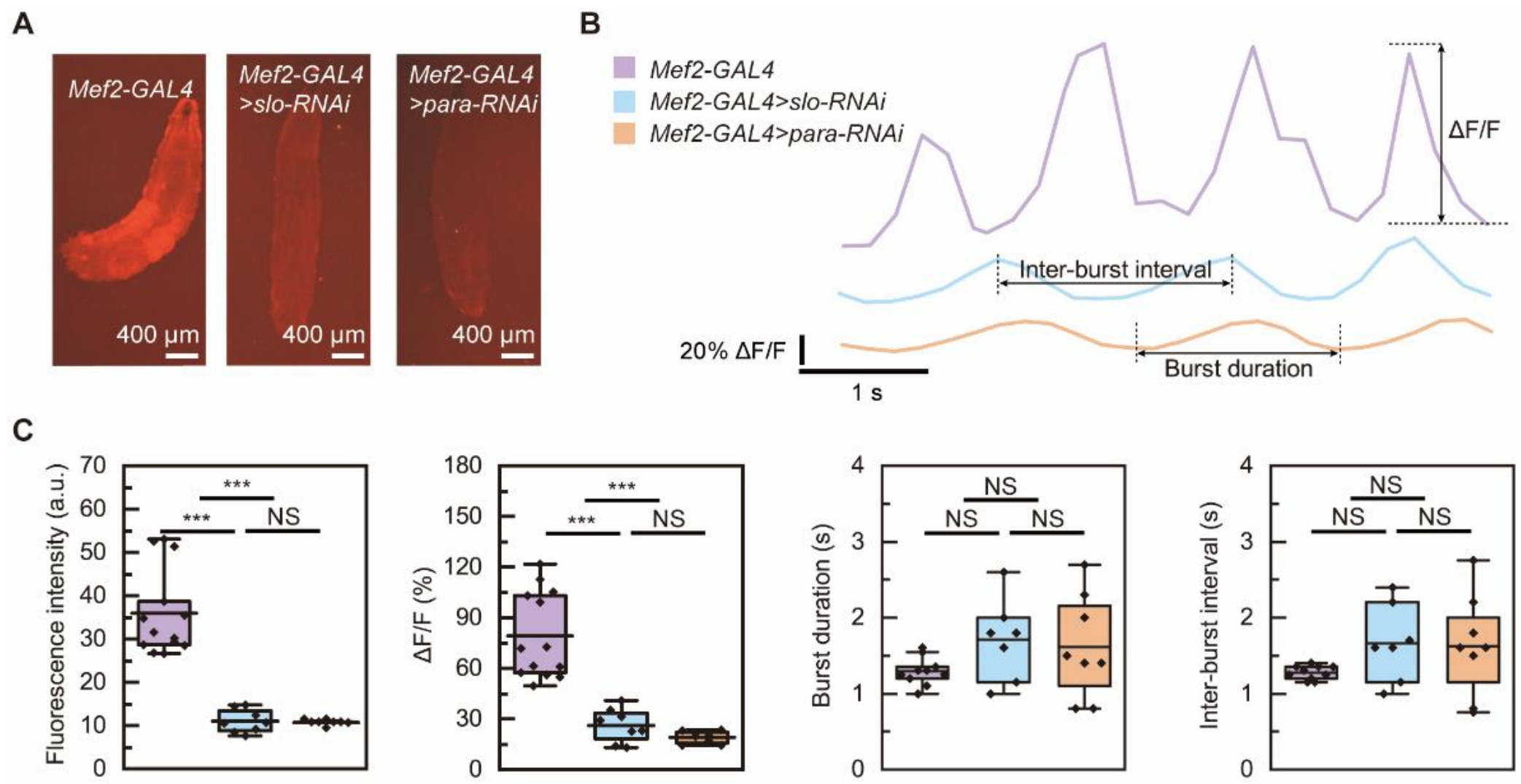

Figure S29. Muscle calcium dynamics in freely moving larvae across three strains. (**A**) Representative calcium imaging frames of freely moving larvae. (**B**) Traces show the real-time muscle calcium responses. (**C**) Statistical results of the three larval strains (n=25).

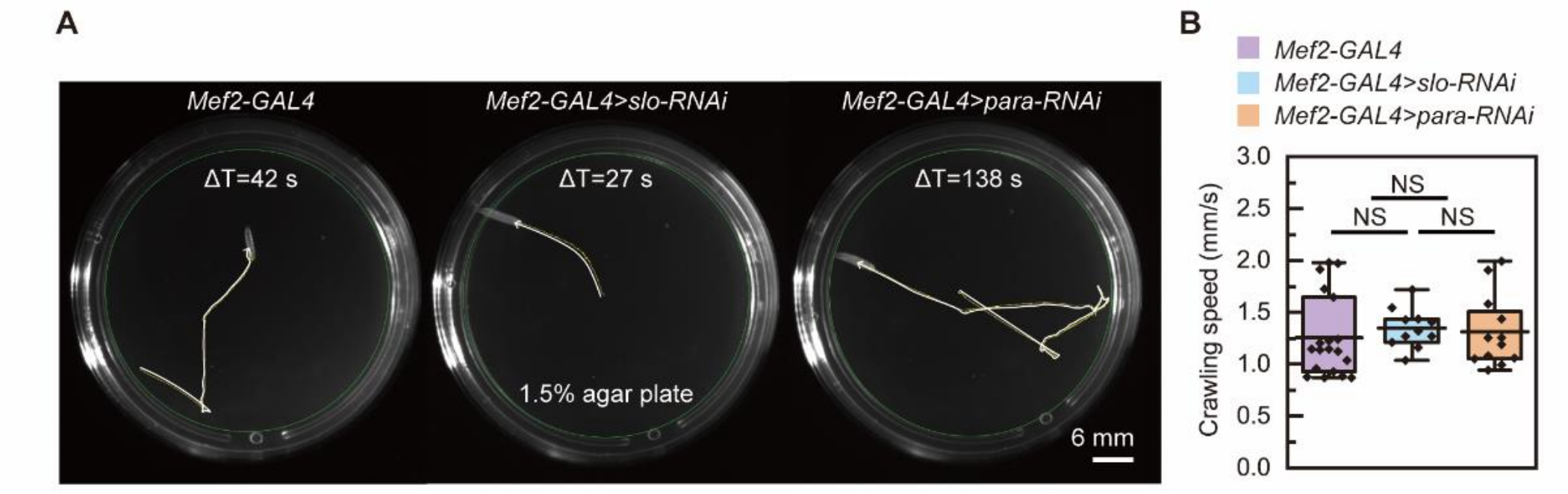

Figure S30. Bright-field behavioral imaging of three larval strains. (**A**) Experimental images of the crawling-trajectory recordings on 1.5% agar plates. (**B**) Statistical results of the crawling speeds (n=25).

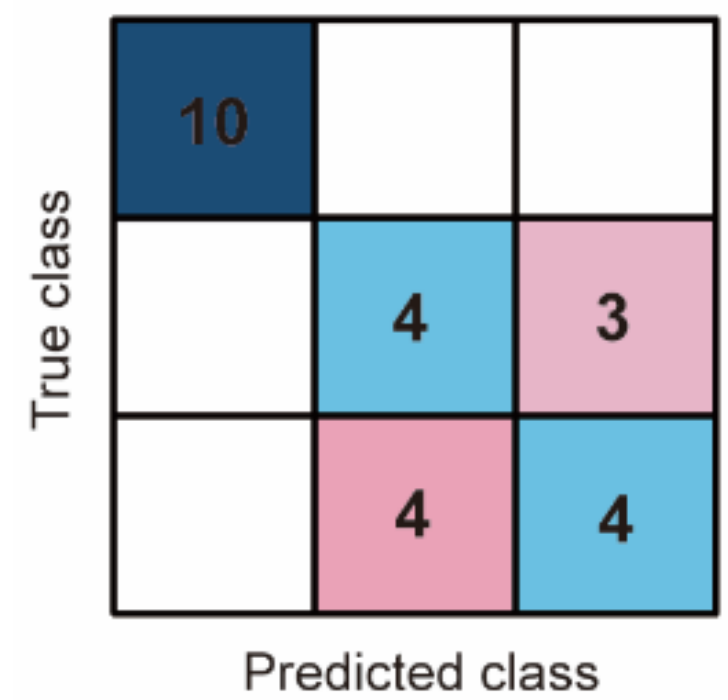

Figure S31. Genotype mapping results based on the benchmarking of camera-based behavior recording methods in free-moving larvae.

Material	Printing parameter	Setting	Base diameter	Tip diameter	Height
Ag	sheath gas: 30 sccm<br>carrier gas: 15 sccm	loop: 39<br>dwell: 1150 µs	~41 µm	~20 µm	~305 µm
Ag	sheath gas: 35 sccm<br>carrier gas: 15 sccm	loop: 22<br>dwell: 770 µs	~31 µm	~15 µm	~193 µm
Ag	sheath gas: 40 sccm<br>carrier gas: 15 sccm	loop: 22<br>dwell: 770 µs	~24 µm	~12 µm	~163 µm
P(VDF-TrFE)	sheath gas: 35 sccm<br>carrier gas: 22 sccm	total time:<br>2 min	~130 µm	~26 µm	~75 µm
P(VDF-TrFE)	sheath gas: 35 sccm<br>carrier gas: 22 sccm	total time:<br>3 min	~143 µm	~44 µm	~118 µm
P(VDF-TrFE)	sheath gas: 40 sccm<br>carrier gas: 25 sccm	total time:<br>3 min	~128 µm	~27 µm	~139 µm
P(VDF-TrFE)	sheath gas: 80 sccm<br>carrier gas: 42 sccm	total time:<br>3 min	~110 µm	~8 µm	~119 µm

Table S1. Relationship between printing parameters and microneedle dimensions.

Movie S1. Bright-field trajectory visualization and calcium imaging of three larval strains.

Movie S2. Larval cuticles indented and rebounded upon contact with two microneedle electrodes.
